# Clinical Characteristics, Diagnosis, and Treatment of Central Nervous System Candidiasis: A Systematic Review of 418 Cases

**DOI:** 10.1007/s11046-026-01097-y

**Published:** 2026-08-19

**Authors:** Guilherme Diogo Silva, Mariane Taborda, Wdson Luis Lima Kruschewsky, Leonardo Torioni, Mariane Cappellini Stricagnolo, Bernardo Ferreira Camilo, Isadora Campos de Sousa Abreu, Isabela Cristina Oliveira Silva, Nadine Correia Silva Santos, Gabriel Diniz Resende, Marcello Mihailenko Chaves Magri, Adriana Satie Gonçalves Kono Magri, Li-Ping Zhu, Vitor Falcão de Oliveira

**Affiliations:** 1 Department of Neurology, Faculdade de Medicina, Hospital das Clinicas HCFMUSP, Universidade de Sao Paulo, Sao Paulo, SP Brazil; 2 Instituto do Câncer do Estado de São Paulo, Universidade de Sao Paulo, Sao Paulo, SP Brazil; 3 Department of Infectious and Parasitic Diseases, Faculdade de Medicina, Hospital das Clinicas HCFMUSP, Universidade de Sao Paulo, Sao Paulo, SP Brazil; 4 Department of Infectious Diseases, Hospital São Paulo, Universidade Federal de São Paulo, Sao Paulo, Brazil; 5 Universidade Municipal de São Caetano Do Sul, Sao Paulo, Brazil; 6 Department of Radiology, Faculdade de Medicina, Hospital das Clinicas HCFMUSP, Universidade de Sao Paulo, Sao Paulo, SP Brazil; 7 Department of Infectious Diseases, National Medical Center for Infectious Diseases, Huashan Hospital, Fudan University, Shanghai, China; 8 Centres for Antimicrobial Optimisation Network (CAMO-Net), Sao Paulo, Brazil

**Keywords:** Candidiasis, Candida, Central nervous system, Systematic review, Neuroimaging

## Abstract

**Background:**

Central nervous system (CNS) candidiasis is a rare but life-threatening complication of invasive candidiasis. However, current knowledge derives mainly from isolated case reports, and no comprehensive synthesis has been previously performed.

**Methods:**

We conducted a systematic review of published cases of CNS candidiasis. PubMed and Embase were searched without language or date restrictions. Eligible cases required microbiological or histopathological confirmation of *Candida* spp. and evidence of CNS involvement on cerebrospinal fluid (CSF) analysis or neuroimaging. Demographic, clinical, radiological, laboratory, therapeutic, and outcome data were extracted. We compared mortality and non-mortality groups, paediatric and adult patients, and diagnostic periods.

**Results:**

We identified 418 patients reported between 1945 and 2024 (median age, 21 years [IQR 0–46]; 62% male). Prior antibiotic use (53%), intravascular catheterization (31%), and neurosurgery (22%) were common risk factors. Neuroimaging was abnormal in 87% of cases, and cerebrospinal fluid was abnormal in 96%, typically showing neutrophilic or lymphocytic pleocytosis and low glucose. Nearly half of cases were pediatric (41% of very-low-birth-weight neonates), who more often had healthcare-associated risk factors and less often immunosuppressive conditions. Overall mortality was 35%, mostly within 30 days; older age was associated with higher mortality, whereas combination antifungal therapy and more recent diagnostic periods were associated with lower mortality in multivariate analysis. Over time, immunosuppression, non-albicans *Candida* species, and antifungal use increased, while autopsy-based diagnoses and mortality declined.

**Conclusion:**

CNS candidiasis is a rare but severe infection with challenging diagnosis and high mortality, emphasizing the importance of early recognition and appropriate antifungal therapy.

**Supplementary Information:**

The online version contains supplementary material available at https://doi.org/10.1007/s11046-026-01097-y.

## Introduction

Candidiasis is an infection caused by *Candida* spp., which represents the leading cause of fungal infections worldwide, with an estimated global burden of 250,000 cases and over 50,000 associated deaths annually [1, 2]. *Candida albicans* remains the most prevalent species causing disease in both adult and pediatric populations. However, non-*albicans* species have become increasingly prominent, particularly in Latin America, Southern Europe, India, and Pakistan [3]. Invasive candidiasis includes both candidemia and deep-seated tissue candidiasis, typically affecting hospitalized patients, especially those who are immunocompromised or critically ill. Common predisposing factors include the presence of central venous catheters, recent surgical procedures (particularly abdominal surgeries with anastomotic leaks), and the use of broad-spectrum antibiotics [2].

Approximately half of patients with disseminated candidiasis develop central nervous system (CNS) involvement, with reported mortality rates ranging from 10 to 50% [4]. CNS invasion by *Candida* species may occur via hematogenous dissemination, particularly in neonates and neutropenic individuals, or through direct inoculation into sterile CNS compartments, such as during neurosurgical procedures or in the presence of intracranial devices. Other risk factors include CARD9 deficiency, chronic corticosteroid use, and intravenous drug use [4].

Current understanding of CNS candidiasis is largely based on case reports and small case series involving diverse patient populations. Notably, many reported cases include coexisting bacterial infections, a frequent finding that may alter both clinical and radiological features, thus complicating diagnostic interpretation. To date, no comprehensive study has systematically aggregated the clinical data of patients with CNS candidiasis, underscoring a significant gap in the literature.

The objective of this study was to perform a systematic review of published cases to synthesize the demographic, clinical, radiological, laboratory, and outcome characteristics of CNS candidiasis. Our data aim to provide a consolidated body of evidence that may inform future clinical guidelines and improve the diagnosis and management of this serious condition.

## Methods

We conducted a systematic literature review in accordance with the recommendations of the Preferred Reporting Items for Systematic Reviews and Meta-Analyses (PRISMA) [5]. We registered the protocol on PROSPERO (CRD42024560522).

### Search Strategy

We searched PubMed/MEDLINE and Embase on July 4th, 2024, using the search terms described in Supplementary data (Online Appendix). We did not use any restriction regarding languages or publication dates.

### Study Selection and Eligibility

Initially, we screened titles and abstracts to evaluate their potential relevance. The titles were independently reviewed by two authors, who selected the article abstracts. Any disagreements were resolved by consulting a third author. Subsequently, we retrieved full-text articles that were deemed relevant, re-assessed their eligibility, and made the final decision to either include or exclude them.

Our inclusion criteria were: 1) documentation of *Candida* using histopathological, microbiological, serological and antigen methods, or molecular techniques by polymerase chain reaction (PCR); AND 2) CNS involvement demonstrated by CNS inflammation [defined as cerebrospinal fluid (CSF) white blood cell count > 5 cells/μL] or neuroimaging abnormalities. Only proven and probable cases were included.

We included case reports and case series, including conference abstracts. Series lacking individual-level data were excluded. Moreover, we excluded articles reporting CNS coinfection, reviews, studies with animals, and in vitro studies. For the analysis of radiological features, articles that did not include cranial magnetic resonance imaging or computed tomography were excluded. Finally, patients whose CNS findings on neuroimaging were attributable to another diagnosis (e.g., CNS vasculitis) were also excluded.

Potential duplicate cases were identified by comparing individual patient-level clinical data and publication characteristics (authorship, institution, and study period). When overlap was confirmed, only the most complete report was included.

### Definitions

Cases were classified as proven when there was a positive CSF culture, positive culture from a CNS abscess or tissue fragment, or compatible histopathological findings. Probable cases required positive CSF biomarkers (e.g., β-D-glucan and mannan) or blood culture for *Candida* species, in association with neurological symptoms, neuroimaging abnormalities, or CSF alterations, and exclusion of other diagnoses of that could explain these findings. Cases classified as possible were excluded from the analysis.

We defined CNS coinfection as the isolation of additional pathogens other than *Candida* from the CNS during the same infectious episode, and disseminated disease as the presence of candidemia associated with CNS involvement or involvement of two non-contiguous anatomical sites. Furthermore, combination therapy was defined as the concomitant use of more than one antifungal agent.

Lastly, adults were defined as individuals aged 18 years or older, and pediatric patients as those younger than 18 years.

### Data Extraction

Data extraction was independently performed by two authors. Any disagreements were resolved by consensus or discussing with a third author. We employed a standardized pilot-tested data collection form that comprised the publication year, country, ages, pediatric variables (newborn, preterm infant, weight, very low birth weight), sex, comorbidities (e.g., haematological malignancy, HIV infection, solid tumors, diabetes mellitus, hematopoietic stem cell transplantation, CARD9 deficiency, and solid organ transplantation), risk factors (e.g., antibiotic previous use, intravascular catheter use, neurosurgery, colonization, total parenteral nutrition, chronic corticosteroids, intravenous drug user, abdominal surgery, and neutropenia), CD4 counts, *Candida* species, neurological manifestations (fever, headache, seizure, altered mental status, vomiting, meningeal signs, focal deficits, and cranial nerve involvement), duration of symptoms, clinical form (disseminated disease or CNS isolated), diagnosis (cell count, predominance of lymphocytes or neutrophils, protein, and glucose in the CSF; CSF direct microscopy; culture; CSF mannan antigen; CSF PCR of *Candida*; CNS histology; and autopsy), surgical treatment (drainage or shunt), antifungal therapy (amphotericin B, flucytosine, fluconazole, voriconazole, echinocandin, and combination therapy), follow-up, and outcomes (death, sequela, and recurrence). The outcomes were assessed at the last follow-up.

Follow-up was defined as the interval from the date of diagnosis or initiation of antifungal therapy to either completion of treatment or the last medical appointment. For cases in which the diagnosis was established at autopsy, follow-up was defined as the period from symptom onset or hospital admission to death. We considered follow-up as the longest period within this interval.

For the calculation of median values, β-D-glucan results reported without an exact numerical value were coded as the corresponding upper reporting limit of the assay (e.g., > 500 pg/mL was recorded as 500 pg/mL).

Case series that included detailed descriptions of each case were extracted as separated case reports. Regarding the collected individual patient data on neuroimaging modalities, we performed and recorded the presence or absence of abnormalities. For a more comprehensive assessment, we included magnetic resonance imaging and/or computed tomography but not cranial X-rays or ultrasounds. Radiological abnormalities were systematically classified into predefined categories: focal lesions, meningeal enhancement, vascular lesions, and ventricular involvement (ventriculitis and/or hydrocephalus). Focal lesions were further characterized by number (solitary vs. multiple), location (supratentorial vs. infratentorial), and presence of ring enhancement consistent with brain abscess. Supratentorial distribution was subclassified by anatomical region, including frontal, parietal, temporal, and occipital lobes, basal ganglia, and thalamus. Meningeal enhancement was categorized as leptomeningeal or pachymeningeal when this distinction was possible. Vascular lesions were recorded as hemorrhagic or ischemic, with infarcts further characterized by laterality (bilateral vs. unilateral) and distribution (nucleocapsular vs. other). Ventricular involvement was noted as present or absent.

Radiological images used for illustrative purposes were obtained from patients with CNS candidiasis identified within our institutional candidemia cohort at the Infectious Diseases Service of Hospital das Clínicas, University of São Paulo. The institutional cohort was approved by the local Research Ethics Committee, which waived the requirement for informed consent because of the retrospective study design. All images were fully de-identified.

### Risk of Bias

Given the heterogeneity of the included studies, methodological quality was assessed qualitatively using the Joanna Briggs Institute (JBI) critical appraisal checklists [6]. The appropriate JBI checklist was applied according to study design (case reports and case series).

### Statistical Analysis

The clinical, radiological, laboratory, treatment data, and outcomes of CNS candidiasis were summarized using total counts and percentages for categorical variables, and median and interquartile range (IQR) for continuous variables.

Due to the descriptive nature of case reports and case series, substantial heterogeneity in variable reporting was observed across studies. For the clinical, diagnostic, and therapeutic characterization of the study population, analyses were performed using complete-case data for each variable. Only cases with available information were included in each analysis, and no data imputation was performed. Therefore, denominators vary according to the number of cases with non-missing data for each variable.

The primary analyses aimed to identify factors associated with mortality by comparing mortality and non-mortality groups. Additional exploratory comparisons were performed between pediatric and adult patients, proven and probable diagnosis, and across diagnostic periods. Categorical variables were analyzed using Pearson’s chi-square test or Fisher’s exact test (depending on the expected cell counts). Continuous variables were analyzed using the Mann–Whitney U test for two-group comparisons (mortality versus non-mortality, proven versus probable diagnosis, and pediatric versus adult patients) and the Kruskal–Wallis test for comparisons across the three diagnostic periods.

We used Multiple Imputation by Chained Equations (MICE) to handle missing data for multivariate analysis to identify independent predictors of mortality. The model for multivariate logistic regression was chosen based on biological plausibility and between-group differences. Variables with structurally missing data due to historical differences in diagnostic availability (e.g., neuroimaging and β-D-glucan) and variables defined or fully observed only after the outcome occurred were excluded from the imputation model. Missing predictor values were imputed using MICE, whereas the outcome (mortality) was not imputed. Regression estimates were pooled using Rubin’s rules. Complete details of the missing data and imputation procedures are provided in the Supplementary Material (Online Appendix).

Furthermore, we represented time-to-event analysis using a Kaplan–Meier curve. We used RStudio 2024.09.1 + 394 for statistical analysis. A *p*-value < 0.05 was considered statistically significant.

## Results

### Demographic Characteristics

The final review included 249 articles (Fig. S1 and Online Appendix in the Supplementary Material). We found 418 patients were diagnosed with CNS candidiasis from 1945 to 2024. The number of cases has surged dramatically since 1975 (Figs. S2, S3). The majority of cases were from the US (n = 148, 35%), followed by China (n = 48, 11%), Brazil (n = 23, 6%), and Taiwan (n = 21, 5%) (Fig. S4).

### Clinical Characteristics

Table 1 summarizes the main clinical characteristics. The median age was 21 years (IQR 0–46), and 62% of the patients were male. Pediatric patients accounted for 46% (190/416) of patients with available age data, of whom 41% (78/190) were newborns (78/416, 19% of all patients). Among the pediatric group, very low birth weight was common, with 61% (59/96) weighing less than 1500 g. The primary comorbidity associated with the disease was immunosuppression, affecting nearly 20% of patients, particularly hematological malignancies (7%), HIV infection (6%), solid tumors (6%), and diabetes mellitus (6%). Among the risk factors for CNS candidiasis, prior antibiotic use was the most common (53%), followed by intravascular catheter use (31%), neurosurgery (22%), colonization (22%), and total parenteral nutrition (17%). The most frequent signs and symptoms included fever (57%), altered mental status (42%), and headache (33%), with symptom duration most often less than one week (39%). Isolated CNS candidiasis was the predominant clinical presentation, accounting for 55% of cases.

**Table 1:** Demographic and clinical characteristics of 418 patients with central nervous system candidiasis, including comparative analyses between adult and pediatric populations

Characteristic	Total n = 418	Adults n = 226	Children n = 190	*p*-value
*Age (years),* median (IQR), n = 416*	21 (0–46)	44 (29–58)	0 (0–1)	** < 0.001**
*Children*	190 †/416§ (46%)	–	–	–
Newborn	–	–	78/190 (41%)	–
Preterm infant (GA < 36 weeks)	–	–	101/118 (86%)	–
Weight (g), median (IQR), n = 86	–	–	1,253 (938–1,772)	–
Very low birth weight (< 1500 g)	–	–	59/96 (61%)	–
*Male*	253/407 (62%)	147/224 (66%)	105/181 (58%)	0.120
*Comorbidities*				
Immunosuppression	77/410 (19%)	61/218 (28%)	16/190 (8%)	** < 0.001**
Haematological malignancy	27/413 (7%)	20/224 (9%)	7/187 (4%)	**0.035**
HIV infection	25/405 (6%)	24/216 (11%)	1/187 (0.5%)	** < 0.001**
*CD4 (/mm*^*3*^*), median (IQR), n* = *21*	52 (20–275)	71 (20–284)	0 (0–0)	0.200
Solid tumors	26/413 (6%)	22/224 (10%)	4/187 (2%)	**0.001**
Diabetes mellitus	25/413 (6%)	25/224 (11%)	0/187 (0%)	** < 0.001**
Hematopoietic stem cell transplantation	12/413 (3%)	11/224 (5%)	1/187 (0.5%)	**0.009**
CARD9 deficiency	9/413 (2%)	6/224 (3%)	3/187 (2%)	0.500
Solid organ transplantation	6/413 (1%)	6/224 (3%)	0/187 (0%)	**0.034**
*Risk factors*				
Antibiotic previous use	191/360 (53%)	79/191 (41%)	112/169 (66%)	** < 0.001**
Intravascular catheter use	99/319 (31%)	29/174 (17%)	70/145 (48%)	** < 0.001**
Neurosurgery	92/413 (22%)	56/224 (25%)	36/187 (19%)	0.200
Colonization	61/272 (22%)	33/142 (23%)	28/130 (22%)	0.700
Total parenteral nutrition	51/293 (17%)	4/156 (3%)	47/137 (34%)	** < 0.001**
Chronic corticosteroid use	58/363 (16%)	47/206 (23%)	11/157 (7%)	** < 0.001**
Intravenous drug user	27/413 (7%)	27/224 (12%)	0/187 (0%)	** < 0.001**
Abdominal surgery	34/413 (8%)	19/224 (8%)	15/187 (8%)	0.900
Neutropenia	15/404 (4%)	7/217 (3%)	8/185 (4%)	0.600
*Signs and symptoms*				
Fever	182/317 (57%)	108/173 (62%)	74/144 (51%)	**0.048**
Altered mental status	131/315 (42%)	92/173 (53%)	39/142 (27%)	** < 0.001**
Headache	103/310 (33%)	88/172 (51%)	15/138 (11%)	** < 0.001**
Vomiting	65/303 (21%)	42/175 (24%)	23/128 (18%)	0.200
Meningeal signs	64/304 (21%)	48/175 (27%)	16/129 (12%)	**0.001**
Focal deficits	64/302 (21%)	53/171 (31%)	11/131 (8%)	** < 0.001**
Seizure	50/305 (16%)	20/174 (11%)	30/131 (23%)	**0.008**
Cranial nerve impairment	33/304 (11%)	30/175 (17%)	3/129 (2%)	** < 0.001**
*Duration of symptoms*, n = 211				
Less than one week	83 (39%)	32/128 (25%)	51/83 (61%)	** < 0.001**
1 to 2 weeks	25 (12%)	17/128 (13%)	8/83 (10%)	0.400
2 to 4 weeks	31 (15%)	24/128 (19%)	7/83 (8%)	**0.039**
4 to 26 weeks	59 (28%)	43/128 (34%)	16/83 (19%)	**0.024**
More than 26 weeks	13 (6%)	12/128 (9%)	1/83 (1%)	**0.016**
*Clinical form,* n = 378				
Isolated CNS	207 (55%)	133/194 (69%)	72/182 (40%)	** < 0.001**
Disseminated disease	171 (45%)	61/189 (32%)	110/182 (60%)	** < 0.001**

IQR: Interquartile range; GA: Gestational age; CNS: Central nervous system. Bold values indicate statistical significance (*p *< 0.05)

^*^Number of cases with available information among all patients for quantitative variables

^†^Number of events with the specified characteristic for qualitative variables

^§^Number of patients in each group with available information for qualitative variables

### Diagnosis Characteristics

The majority of diagnoses of CNS candidiasis were proven (88%), with CSF culture being the most important method, showing 74% of positivity (Table 2). Blood cultures were positive in 44% of cases. *Candida albicans* (66%) was the most frequently isolated species, followed by *C. tropicali*s (8%) and *C. parapsilosis* (6%) (Fig. S5). In 10% of the samples, the species were not identified. β-D-glucan testing in CSF was positive in 18 patients (95%), with a median value of 500 pg/mL (range: 185–1,158). Most patients presented CSF abnormalities (96%), including increased cellularity (90%), with both neutrophilic (57%) and lymphocytic (43%) patterns, as well as elevated protein and decreased glucose levels. Histology was positive in 88% of cases. Lastly, the autopsy was performed in 20% of patients, and nearly half of these were diagnosed at autopsy.

**Table 2:** Diagnosis characteristics of 418 patients with central nervous system candidiasis, including comparative analyses between adult and pediatric populations

Characteristic	Total n = 418	Adults n = 226	Children n = 190	*p*-value
*Proven diagnosis*	367†/418§ (88%)	208/226 (92%)	157/190 (83%)	**0.004**
*Blood*				
Positive blood cultures	116/261 (44%)	32/126 (25%)	84/135 (62%)	** < 0.001**
*Cerebrospinal fluid*				
Positive direct microscopy	72/139 (52%)	37/79 (47%)	34/58 (59%)	0.200
Positive culture	251/339 (74%)	123/168 (73%)	126/169 (75%)	0.800
Positive ß-D-Glucan	18/19 (95%)	9/9 (100%)	9/10 (90%)	> 0.900
*ß-D-Glucan* values *(pg/mL), median (IQR), n* = 17*	500 (185–1,158)	500 (500–4,262)	361 (152–600)	0.200
Positive mannan antigen	11/15 (73%)	8/11 (73%)	3/4 (75%)	> 0.900
Positive PCR	16/20 (80%)	14/18 (78%)	2/2 (100%)	> 0.900
Any CSF abnormality	295/308 (96%)	145/154 (94%)	148/152 (97%)	0.200
Leukocytes > 5 cells	240/267 (90%)	121/131 (92%)	117/134 (87%)	0.200
*Leucocytes (cels/mm*^*3*^*), median (IQR), n* = 253*	174 (37–590)	230 (60–775)	110 (22–399)	**0.003**
Lymphocytic predominance	64/148 (43%)	37/90 (41%)	26/56 (46%)	0.500
*Lymphocytic (%), median (IQR), n* = 125*	33 (12–68)	35 (15–75)	36 (10–60)	0.300
Neutrophil predominance	84/147 (57%)	54/89 (61%)	29/56 (52%)	0.300
*Neutrophil (%), median (IQR), n* = 124*	55 (18–79)	59 (22–80)	46 (13–70)	0.100
Protein > 50 mg/dl	230/254 (91%)	116/128 (91%)	112/124 (90%)	> 0.900
*Protein (mg/dl), median (IQR), n* = 246*	150 (80–288)	139 (78–341)	155 (84–248)	0.500
Glucose < 40 mg/dl	135/230 (59%)	63/118 (53%)	71/110 (65%)	0.087
*Glucose (mg/dl), median (IQR), n* = 219*	33 (21–51)	38 (24–57)	31 (19–45)	**0.016**
*Positive histology*	115/131 (88%)	81/91 (89%)	34/40 (85%)	0.500
Positive biopsy culture	39/153 (25%)	28/98 (29%)	11/55 (20%)	0.200
*Autopsy*	80/396 (20%)	48/188 (26%)	32/167 (19%)	0.200
Diagnosis by autopsy	37/80 (46%)	16/206 (8%)	21/187 (11%)	0.200

IQR: Interquartile range; CSF: cerebrospinal fluid; PCR: Polymerase chain reaction. Bold values indicate statistical significance (*p *< 0.05)

^*^Number of cases with available information among all patients for quantitative variables

^†^Number of events with the specified characteristic for qualitative variables

^§^Number of patients in each group with available information for qualitative variables

Among patients with proven CNS candidiasis, blood cultures were positive in 37% of cases, whereas CSF cultures were positive in 84%. CSF mannan antigen testing was positive in 75% of cases, and CSF PCR was positive in 78%. β-D-glucan was measured in only 12 patients, all of whom had positive results. Histopathology was positive in 91% of cases. Compared with probable cases, patients with proven CNS candidiasis were less likely to have CSF glucose levels < 40 mg/dL (*p *= 0.007). The remaining CSF biochemical parameters were similar between the groups (Table S1).

### Radiological Characteristics

**Fig. 1:**
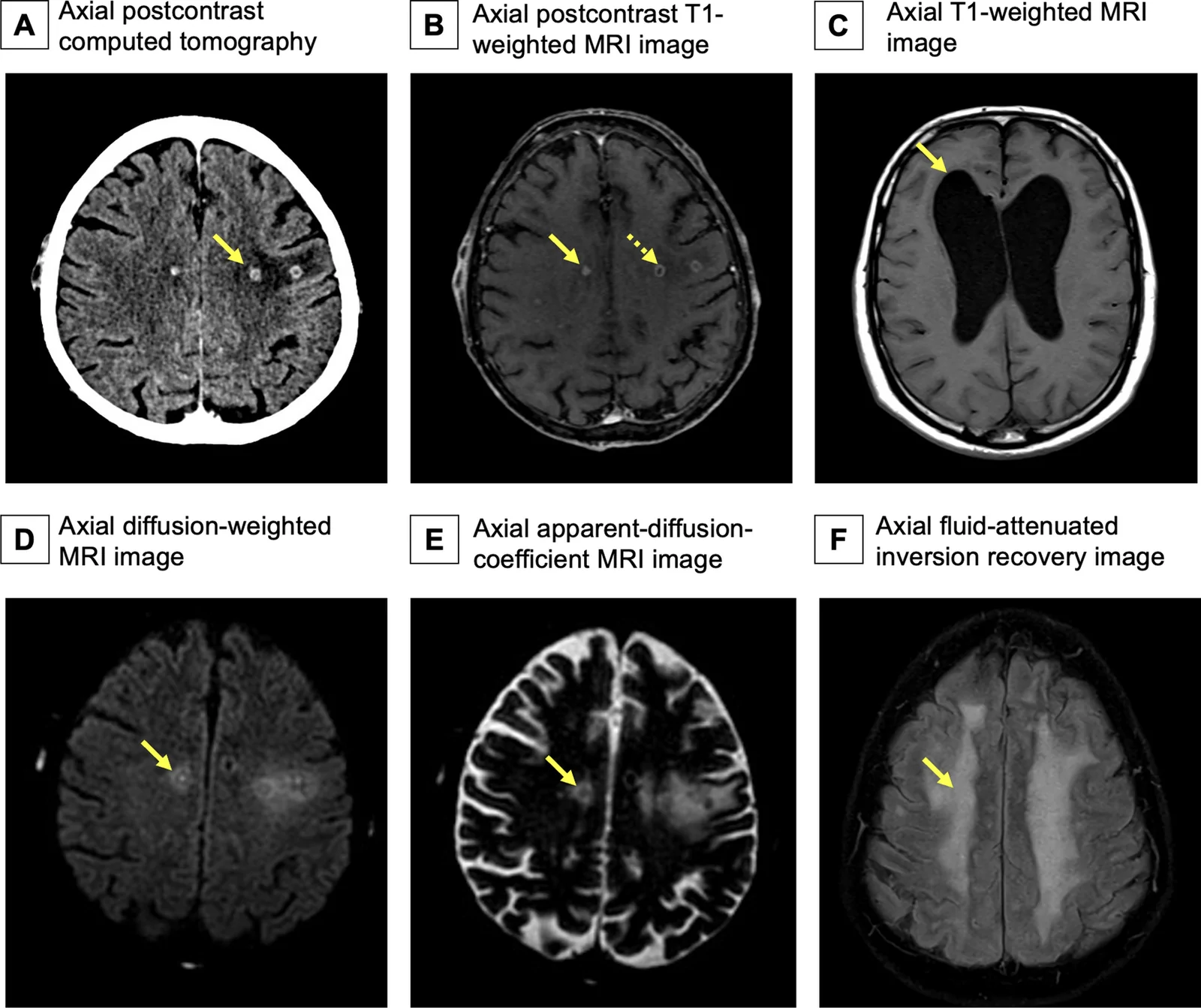
Neuroimaging findings in central nervous system candidiasis. Axial postcontrast computed tomography (**A**) and T1-weighted magnetic resonance (**B**) images show small nodular and ring-enhancing lesions in the frontal lobe. Axial diffusion-weighted imaging (**D**) and apparent-diffusion-coefficient (**E**) show restricted diffusion consistent with brain abscess. An axial T1-weighted magnetic resonance image shows hydrocephalus (**C**). An axial fluid-attenuated inversion recovery (FLAIR) image demonstrates bilateral white matter hyperintensities, consistent with leukoencephalopathy (**F**)

Table 3 summarizes the radiological features of 140 patients with CNS. Neuroimaging was performed using magnetic resonance imaging in 97 of 140 patients (69%) and computed tomography in 82 of 140 patients (59%). Abnormal findings on neuroimaging were observed in 122 of 140 patients (87%). The most common abnormalities were focal lesions, predominantly appearing as ring-enhancing brain abscesses, typically located in the supratentorial region, especially in the frontal and parietal lobes and basal ganglia. Focal lesions were multiple in more than half of the cases. The second most frequent neuroimaging pattern was ventricular involvement, identified in over one-third of patients. Meningeal enhancement was reported in approximately one-quarter of cases, with a leptomeningeal pattern observed in the vast majority. Vascular lesions included both hemorrhagic and ischemic events. Brain infarctions were often bilateral and involved the basal ganglia in about one-third of cases. Less common neuroimaging findings included cerebral artery aneurysms (n = 5) and progressive leukoencephalopathy (n = 3). Figure 1 illustrates the neuroimaging findings of CNS candidiasis. Among the 57 patients with radiological follow-up, improvement was observed in 50 cases (88%).

**Table 3:** Radiological characteristics of patients with central nervous system candidiasis, including comparative analyses between adult and pediatric populations

Characteristic	Total n = 418	Adults n = 226	Children n = 190	*p*-value
*Focal lesions*	62†/140§ (44%)	33/87 (38%)	29/53 (55%)	0.052
Brain abscess	48/62 (77%)	26/33 (79%)	22/29 (76%)	0.800
Multiple lesions	36/62 (58%)	17/33 (52%)	19/29 (66%)	0.300
Supratentorial lesions	50/62 (81%)	25/33 (76%)	25/29 (86%)	0.400
*Frontal lobe*	15/44 (34%)	8/27 (30%)	7/17 (41%)	0.400
*Temporal lobe*	10/44 (23%)	8/27 (30%)	2/17 (12%)	0.300
*Parietal lobe*	13/44 (30%)	9/27 (33%)	4/17 (24%)	0.700
*Occipital lobe*	8/44 (18%)	4/27 (15%)	4/17 (24%)	0.700
*Basal ganglia*	12/44 (27%)	7/27 (26%)	5/19 (26%)	> 0.900
*Thalamus*	4/44 (9%)	3/27 (11%)	1/17 (6%)	> 0.900
*Meningeal enhancement*	34/140 (24%)	30/87 (34%)	4/53 (8%)	** < 0.001**
Leptomeningeal enhancement	25/31 (81%)	24/29 (83%)	1/2 (50%)	0.400
*Vascular lesions*	18/140 (13%)	15/87 (17%)	3/53 (6%)	**0.047**
Hemorrhage	10/18 (56%)	7/15 (47%)	3/3 (100%)	0.200
Brain infarcts	14/18 (78%)	13/15 (87%)	1/3 (33%)	0.110
*Bilateral infarcts*	8/18 (44%)	8/15 (53%)	0/3 (0%)	0.200
*Nucleocapsular infarcts*	5/18 (28%)	5/15 (33%)	0/3 (0%)	0.500
*Ventricular involvement*	49/140 (35%)	28/87 (32%)	21/53 (40%)	0.400

^†^Number of events with the specified characteristic for qualitative variables. Bold values indicate statistical significance (*p *< 0.05)

^§^Number of patients in each group with available information for qualitative variables

### Therapeutic Characteristics

Neurosurgical treatment was performed in 39% of patients, with shunting (25%) being more frequent than abscess drainage (13%) (Table 4). Regarding antifungal therapy, nearly 90% of patients received treatment, with amphotericin B being the most commonly used agent (70%), of which 41% received the deoxycholate formulation, followed by fluconazole (43%) and flucytosine (39%). Combination therapy was a prevalent treatment approach, administered to 47% of patients, predominantly consisting of amphotericin B plus flucytosine in 33%. The overall median follow-up duration was four months (2–12). Among autopsy cases, the median follow-up was similar, at three months (1–10).

**Table 4:** Treatment and outcomes characteristics of 418 patients with central nervous system candidiasis, with comparisons between adult and pediatric patients

Characteristic	Total n = 418	Adults n = 226	Children n = 190	*p*-value
*Neurosurgical treatment*	146†/376§ (39%)	92 (48%)	54 (30%)	** < 0.001**
Drainage	49/366 (13%)	38/191 (20%)	11/173 (6%)	** < 0.001**
Shunt	91/366 (25%)	54/191 (28%)	37/173 (21%)	0.130
*Antifungal treatment*	335/378 (89%)	173/201 (86%)	162/177 (92%)	0.10
Amphotericin B	253/364 (70%)	127/191 (66%)	126/173 (73%)	0.2
*Deoxycholate*	133/325 (41%)	52/174 (30%)	81/151 (54%)	** < 0.001**
*Liposomal*	72/322 (22%)	50/172 (29%)	22/150 (15%)	**0.002**
Fluconazole	154/362 (43%)	92/189 (49%)	62/173 (36%)	**0.014**
Flucytosine	142/362 (39%)	74/189 (39%)	68/173 (39%)	> 0.900
Voriconazole	44/362 (12%)	27/189 (14%)	17/173 (10%)	0.200
Echinocandin	27/362 (7%)	17/189 (9%)	10/173 (6%)	0.130
Intrathecal amphotericin B	20/413 (5%)	8/221 (4%)	12/190 (6%)	0.200
Combination therapy	171/362 (47%)	90/189 (48%)	81/173 (47%)	0.900
*Amphotericin B plus flucytosine*	116/349 (33%)	61/184 (33%)	55/165 (33%)	> 0.900
*Amphotericin B plus fluconazole*	27/349 (8%)	19/185 (10%)	8/165 (5%)	0.058
*Follow-up,* median (months), n = 239*	4 (2–12)	4 (2–12)	4 (2–12)	0.9
*Death*	143/403 (35%)	98/223 (44%)	45/180 (25%)	** < 0.001**
*Sequela*	36/210 (17%)	21/107 (20%)	15/103 (15%)	0.300
*Recurrence*	21/208 (10%)	13/97 (13%)	8/111 (7%)	0.14

IQR: Interquartile range. Bold values indicate statistical significance (*p *< 0.05)

^*^Number of cases with available information among all patients for quantitative variables

^†^Number of events with the specified characteristic for qualitative variables

^§^Number of patients in each group with available information for qualitative variables

### Outcomes

**Fig. 2:**
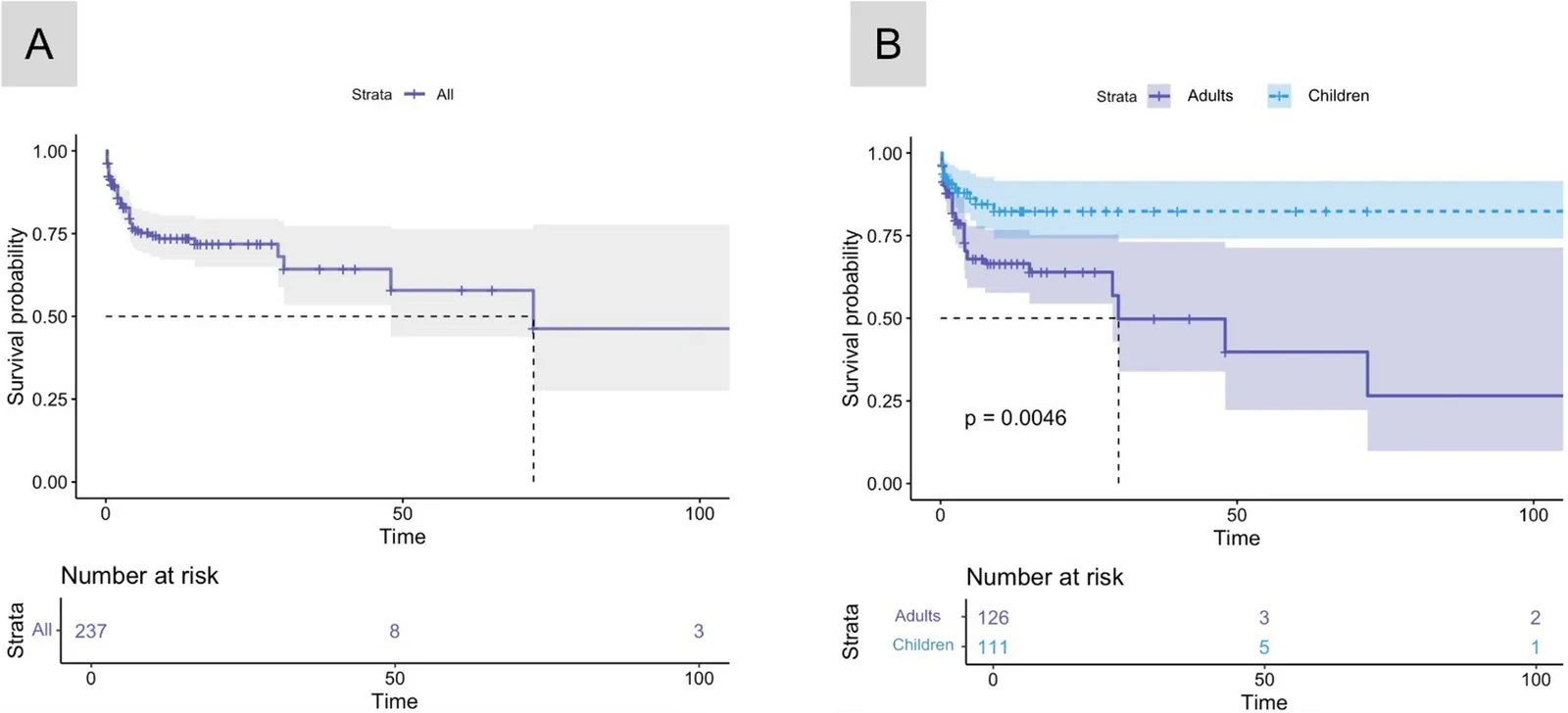
Kaplan–Meier curves for mortality in central nervous system candidiasis. **A** Overall mortality. **B** Comparison between adults and children

The overall mortality was 35% (143/403), particularly in the first 30 days after the diagnosis of CNS candidiasis (Fig. 2A). The median year of publication associated with non-survivor patients was significantly lower than that observed among survivor patients (1,992 vs. 2,004; *p *< 0.001) (Table 5). After excluding cases diagnosed by autopsy, mortality remained high at 29% (106/366) (Fig. S6).

**Table 5:** Comparison between survivor and non-survivor groups of patients with central nervous system candidiasis for variables associated with mortality by multivariate analysis

Characteristics N = 403	Survivor N = 260	Non-survivor N = 143	*p*-value	Multivariate analysis		
				OR	95% CI	*p*-value
Median year of publication, years (IQR)	2,004 (1,994–2,015)	1,992 (1,977–2,008)	** < 0.001**	0.62	0.52–0.72	** < 0.001**
Median age, years (IQR), n = 401*	8 (0–41)	32 (0–52)	** < 0.001**	1.02	1.01–1.03	** < 0.001**
Children	135†/260§ (52%)	45/143 (31%)	** < 0.001**			
Newborn	52/140 (37%)	24/47 (51%)	0.093			
Male	156/250 (62%)	89/142 (63%)	> 0.9	1.12	0.68–1.85	0.660
*Comorbidities and risk factors*						
Immunosuppression	44/252 (17%)	33/143 (23%)	0.2			
Haematological malignancy	10/256 (4%)	17/142 (12%)	**0.002**	1.68	0.54–5.19	0.370
Previous neurosurgery	72/256 (28%)	18/142 (13%)	** < 0.001**	0.52	0.26–1.02	0.060
Diabetes mellitus	14/256 (5%)	11/142 (8%)	0.4			
CARD9 deficiency	7/256 (3%)	2/142 (1%)	0.5			
HIV infection	12/248 (5%)	13/142 (9%)	0.094			
Chronic steroid use	34/228 (15%)	24/127 (19%)	0.3			
Immunosuppressive drugs	17/249 (7%)	20/140 (14%)	**0.016**	1.7	0.63–4.59	0.295
*Signs and symptoms*						
Fever	119/211 (56%)	57/99 (58%)	0.8			
Headache	70/204 (34%)	32/99 (32%)	0.6			
Altered mental status	75/208 (36%)	54/100 (54%)	**0.003**	1.5	0.86–2.61	0.150
Meningeal irritation signs	35/205 (17%)	28/92 (30%)	**0.009**	1.21	0.60–2.46	0.595
Seizure	33/205 (16%)	15/93 (16%)	> 0.9			
Symptoms onset median, weeks (IQR)	2 (1–6)	3 (1–8)	0.12			
*Radiological Findings*						
Meningeal enhancement	25/102 (25%)	8/33 (24%)	> 0.9			
Focal lesion	49/102 (48%)	10/33 (30%)	0.074			
Vasculitis	10/102 (10%)	7/33 (21%)	0.086			
Ventricular involvement	31/102 (30%)	17/33 (52%)	**0.028**			
*Mycological Diagnosis*						
Positive blood cultures	75/173 (43%)	37/83 (45%)	0.9			
Positive CSF culture	172/223 (77%)	68/102 (67%)	**0.046**	0.68	0.35–1.30	0.238
Positive serum BDG	16/20 (80%)	3/4 (75%)	> 0.9			
Positive CSF BDG	16/17 (94%)	2/2 (100%)	> 0.9			
Any CSF abnormality	200/211 (95%)	81/83 (98%)	0.4			
*Treatment*						
Median follow-up, months (IQR), n = 237*	6 (2–12)	2 (0–4)	** < 0.001**			
Neurosurgery	99/242 (41%)	43/119 (36%)	0.4			
Antifungals	254/259 (98%)	77/115 (67%)	** < 0.001**			
Amphotericin B	195/256 (76%)	56/104 (54%)	** < 0.001**			
Liposomal Amphotericin B	58/221 (26%)	13/97 (13%)	**0.011**			
Fluconazole	126/256 (49%)	25/102 (25%)	** < 0.001**			
Flucytosine	113/256 (44%)	27/102 (26%)	**0.002**			
Combination therapy	132/256 (52%)	37/102 (36%)	**0.009**	0.51	0.30–0.88	**0.015**

IQR: Interquartile range; CSF: Cerebrospinal fluid. Bold values indicate statistical significance (*p *< 0.05)

^*^Number of cases with available information among all patients for quantitative variables

^†^Number of events with the specified characteristic for qualitative variables

^§^Number of patients in each group with available information for qualitative variables

Patients who survived were generally younger (8 vs. 32 years; *p *< 0.001), comprised a greater proportion of pediatric cases (52% vs. 31%; *p *< 0.001), presented with fewer hematological malignancies (4% vs. 12%; *p *= 0.002), were more likely to have undergone previous neurosurgery (28% vs. 13%; *p *< 0.001), and were less frequently treated with immunosuppressive drugs (7% vs. 14%; *p *= 0.016).

In terms of clinical presentation, non-survivor patients exhibited altered mental status (54% vs. 36%) and meningeal irritation signs (30% vs. 17%) significantly more often than survivors. Ventricular involvement was the only radiological findings that showed a statistically significant difference, being more frequent among non-survivor patients (52% vs. 30%; *p *= 0.028). Positive CSF cultures were less frequent among non-survivor patients (67% vs. 77%; *p *= 0.046).

The median follow-up duration was shorter in patients who died (2 months vs. 6 months; *p *< 0.001). Furthermore, these patients received fewer antifungal agents overall and were less frequently treated with combination therapy (36% vs. 52%; *p *= 0.009) (Table 5). Additional comparisons are presented in Table S2.

After multiple imputation, the multivariable model identified the following independent associations with mortality: age (OR 1.02, 95% CI 1.01–1.03, *p *< 0.001), combination therapy (OR 0.51, 95% CI 0.30–0.88, *p *= 0.015), and diagnosis period (OR 0.62, 95% CI 0.52–0.72, *p *< 0.001) (Table 5). Effect estimates from the complete-case sensitivity analysis were directionally consistent with the multiple-imputation model, although the association between combination therapy and mortality was no longer statistically significant (Table S3).

### Comparison between Adults and Children

Age information was unavailable for two patients. Adults had a higher prevalence of immunosuppressive conditions (28% vs. 8%, *p *< 0.001) (Table 1). In contrast, children exhibited more risk factors, particularly total parenteral nutrition (34% vs. 3%, *p *< 0.001), prior antibiotic use (66% vs. 41%, *p *< 0.001), and intravascular catheter use (48% vs. 17%, *p *< 0.001).

Children also showed fewer statistically significant clinical signs and symptoms, such as fever (*p *= 0.048), headache (*p *< 0.001), altered mental status (*p *< 0.001), meningeal signs (*p *= 0.001), and focal deficits (*p *< 0.001). Symptom duration was more acute in pediatric patients, with most presenting for less than one week (61% vs. 25%), whereas adults more frequently had chronic symptoms, especially those lasting more than four weeks. Additionally, disseminated disease was more common among children (60% vs. 32%, *p *< 0.001).

Regarding the diagnostic parameters, the most notable difference was blood culture positivity, which was higher in children (62% vs. 25%, *p *< 0.001) (Table 2). For the other parameters, the groups were essentially similar, with no statistically significant differences.

The radiology findings were also very similar (Table 3). However, children had lower rates of meningeal enhancement (8% vs. 34%, *p *< 0.001) and vascular lesions (6% vs. 17%, *p *= 0.047).

Lastly, children underwent fewer surgical procedures (*p *< 0.001), particularly drainage, were more frequently treated with amphotericin B deoxycholate (*p *< 0.001), and received fluconazole less often (*p *= 0.014) (Table 4). Radiological follow-up in children more frequently showed stable disease or improvement. The mortality was especially high among adults (*p *= 0.0046) (Fig. 2B).

### Comparison of Characteristics by Diagnostic Period

The 80-year study period spans major changes that could have influenced the observed clinical and diagnostic characteristics. Therefore, we analysed three diagnostic periods: 1945–1980 (period 1), 1981–2000 (period 2), and 2001–2024 (period 3) (Table S4). Paediatric cases were more frequent in period 2 (58%). The proportion of immunosuppressed patients increased in the more recent decades, accounting for 21% of cases in both periods 2 and 3, compared with approximately 8% in period 1.

Previous neurosurgery as a risk factor showed a progressive increase over time, with higher proportions observed in period 2 (23%) and period 3 (28%). The duration of symptoms was similar across the three periods (*p *= 0.7). Isolated CNS candidiasis became more frequent over time, particularly in period 3 (58%) and period 2 (56%), compared with period 1 (38%).

Probable cases were more commonly identified in period 3 (22%). All positive CSF β-D-glucan results were observed in period 3. The proportion of positive CSF cultures was higher in periods 2 and 3 (87% and 71%, respectively; *p *< 0.001). Diagnoses established at autopsy decreased markedly over time, from 22% in period 1 to 7.2% in period 2 and 6.8% in period 3. A progressive increase in non-*albicans Candida* species was observed, particularly *Candida parapsilosis* (10%), *Candida tropicalis* (7.6%), and *Candida glabrata* (5.6%) in period 3.

Regarding management, the proportion of patients undergoing neurosurgical intervention was similar across the three periods (*p *= 0.6). In contrast, antifungal therapy was more frequently administered in the more recent periods, with treatment rates of 91% in period 2 and 96% in period 3, whereas fewer than half of patients received antifungal therapy in period 1. Among treated patients, liposomal amphotericin B was more commonly used in period 3 (41%), along with azoles, particularly fluconazole (67%), and other antifungal classes, such as echinocandins (14%). Flucytosine use was more frequent in period 2 (56%), and combination antifungal therapy was also more commonly employed during this period.

The median follow-up duration was similar across diagnostic periods (*p *= 0.3). However, mortality decreased significantly over time (*p *< 0.001), from 78% in period 1 to 33% in period 2 and 23% in period 3.

### Risk of Bias

Among the 213 case reports, the most frequent methodological limitation was inadequate reporting of adverse or unanticipated events (JBI item 7), which was rated as "No" or "Unclear" in 50 studies (23.5%). However, this item was not considered a major concern for the present review because adverse events were not among the outcomes of interest. Among the 36 case series, the most common methodological limitation was unclear reporting of consecutive participant inclusion (JBI item 4; 15/36, 41.7%), primarily in small case series. Detailed quality assessments for each included study are provided in Table S5.

## Discussion

This systematic review demonstrates that CNS candidiasis is a severe neuroinfection with a male predominance and two distinct demographic profiles: neonates and immunosuppressed adults. The typical clinical presentation consists of an acute onset of headache, fever, and altered mental status in patients with risk factors such as immunosuppression (e.g., hematological malignancy or CARD9 mutation) or exposure to iatrogenic factors, including neurosurgery and intravascular catheter use. The most frequent radiological findings were multiple brain abscesses and ventricular involvement, while cerebrospinal fluid analysis consistently showed pleocytosis with neutrophilic or lymphocytic patterns. Mortality remained high at 35%, particularly among older patients, but decreased over the last decades and was lower in patients who received combination antifungal therapy in the multivariable analysis.

The most common presentations of CNS candidiasis was a subacute onset of fever, headache, and altered mental state [7, 8]. Cerebrospinal fluid analysis typically revealed pleocytosis with neutrophil predominance and low glucose levels, findings that may overlap with those of bacterial meningitis and therefore warrant careful interpretation to avoid misdiagnosis.

The limited sensitivity of culture makes the diagnosis of CNS candidiasis particularly challenging [6]. In our study, the moderate positivity observed in CSF cultures likely reflects the predominance of confirmed cases in which CSF sampling was systematically performed. In contrast, blood cultures, although frequently obtained in the analyzed cases, showed low sensitivity, with positivity in fewer than 50% of patients, as expected. Other diagnostic methods, such as mannan and PCR, were not widely available. What is noteworthy is that many cases were identified through autopsy studies, highlighting the difficulty of establishing the diagnosis [9].

Radiological features of central nervous system candidiasis overlap with those reported in other fungal CNS infections, typically including focal lesions, meningeal enhancement, brain infarcts, and ventricular involvement. Compared with cryptococcal infection, CNS candidiasis was associated with a higher frequency of focal lesions (44% vs. 15%), most commonly presenting as multiple ring-enhancing abscesses. In contrast, cryptococcosis showed a markedly higher rate of meningeal enhancement (87% vs. 24%) and was uniquely characterized by the presence of pseudocysts [10]. Sporotrichosis also predominantly manifested with meningeal imaging findings (49% vs. 24%) rather than parenchymal lesions (18% vs. 44%), which were more typical of candidiasis [11]. Although focal lesions were the most common imaging finding in CNS candidiasis, their frequency was lower than in neuroparacoccidioidomycosis (44% vs. 91%), where large pseudotumoral lesions are the hallmark [12]. CNS histoplasmosis and CNS candidiasis showed comparable frequencies of focal lesions, ventricular involvement, and leptomeningeal enhancement [13].

According to current guidelines, liposomal amphotericin B, usually in combination with 5-flucytosine, is strongly recommended for the treatment of CNS candidiasis [1]. However, no comparative studies have established the optimal therapeutic regimen, largely due to the rarity of this disease. Both deoxycholate amphotericin B and liposomal amphotericin B showed the greatest efficacy against *Candida albicans* in brain tissue. The liposomal formulation achieves higher brain concentrations and is associated with lower nephrotoxicity, allowing higher doses [14, 15]. In our systematic review, we found that combination antifungal therapy, particularly with amphotericin B and 5-flucytosine, was associated with reduced mortality in the multivariable analysis; however, this association was not retained in the complete-case sensitivity analysis.

Increased mortality was associated with older age, corticosteroid use, and inadequate therapeutic management [16]. In this study, mortality decreased markedly over time, likely reflecting multiple factors, including changes in case ascertainment, patient characteristics, diagnostic practices, availability of antifungal therapy, and the decreasing proportion of cases diagnosed only at autopsy. This is the first study to address the factors associated with mortality in CNS candidiasis.

This study has some limitations. First, we included only case reports and case series with individual-level data. Although this approach allows a more precise assessment of mortality predictors, it is accompanied by substantial heterogeneity in the reporting of clinical, radiological, and laboratory variables, resulting in a high proportion of missing data. Second, publication bias and the incorporation of microbiological and histopathological findings into the diagnostic definitions may have inflated the observed positivity rates of some diagnostic tests, particularly CSF culture. Third, the short median follow-up limits conclusions about long-term outcomes, and the time origin was not standardized across patients, reflecting the inherent limitations of synthesizing published case reports and case series. An additional limitation is that the duration of antifungal therapy was inconsistently reported. In addition, a subset of cases was derived from autopsy studies, which often lacked detailed clinical information and may have overestimated mortality; however, mortality remained high even after exclusion of these cases. Lastly, although our search strategy retrieved more than 6,500 records, the absence of some historical fungal nomenclature, particularly in older case reports, and alternative terminology for CNS involvement may have reduced its sensitivity for identifying some eligible reports. Despite these limitations, this review compiles data from a large number of patients with a rare condition and provides clinically relevant insights into the presentation, diagnostic work-up, management, and outcomes of CNS candidiasis. Future prospective studies are needed to improve data completeness and to confirm the risk factors identified in this study.

## Conclusion

CNS candidiasis is a rare but severe infection, predominantly affecting neonates and immunosuppressed adults, with a subacute presentation that often mimics bacterial meningitis. Diagnosis remains difficult due to nonspecific CSF findings and limited culture sensitivity, while neuroimaging commonly shows multiple abscesses and ventricular involvement. Mortality remains high, emphasizing the importance of early recognition and appropriate management. Further studies are needed to evaluate the role of combination antifungal therapy in improving clinical outcomes.

## Supplementary Information

Below is the link to the electronic supplementary material.

Additional references are available in the Supplementary Material (Online Appendix).
Supplementary file1 (DOCX 5082 kb)

## Data Availability

All data supporting the findings of this study are available within the paper and its supplementary material.
