# Supplementary material for: Clinical Characteristics, Diagnosis, and Treatment of Central Nervous System Candidiasis: A Systematic Review of 418 Cases
Source: Mycopathologia. 2026 Aug 19;191(5):73. doi: 10.1007/s11046-026-01097-y (PMC13490001; doi:10.1007/s11046-026-01097-y)
Supplement: Supplementary file 1 [file 11046_2026_1097_MOESM1_ESM.docx]

**Supplementary Material**

**Silva GD *et al*.** Clinical characteristics, diagnosis, and treatment of central nervous system candidiasis: a systematic review of 418 cases

| Figure S1 | Page 2 |
| --- | --- |
| Figure S2 | Page 3 |
| Figure S3 | Page 4 |
| Figure S4 | Page 5 |
| Figure S5 | Page 6 |
| Figure S6 | Pages 7 |
| Table S1 | Pages 8-9 |
| Table S2 | Pages 10-13 |
| Table S3 | Pages 14 |
| Table S4 | Pages 15-19 |
| Table S5 | Pages 20-30 |
| Appendix | Pages 31-61 |

**Figure S1**. Study selection for systematic review of cases of central nervous system candidiasis

**Figure S2**. Cumulative number of cases with central nervous system candidiasis by year

**Figure S3**. Number of cases with central nervous system candidiasis by year

**Figure S4.** Geographic distribution of the country of publication of patients with central nervous system candidiasis from 1945 to 2024

**Figure S5.** Proportion of *Candida* species among patients with central nervous system candidiasis

**Figure S6.** Kaplan–Meier curves for mortality in central nervous system candidiasis after exclusion of cases with an autopsy-established diagnosis

**Table S1.** Comparison of laboratory characteristics between patients with proven and probable central nervous system candidiasis

| **Characteristic** | **Probable**  n = 51 | **Proven**  n = 367 | **p-value** |
| --- | --- | --- | --- |
| **Blood** |  |  |  |
| Positive blood cultures | 36/46 (78%) | 80/215 (37%) | **< 0.001** |
| **Cerebrospinal fluid** |  |  |  |
| Positive direct microscopy | 0/9 (0%) | 72/130 (55%) | **< 0.001** |
| Positive culture | 0/41 (0%) | 251/298 (84%) | **< 0.001** |
| Positive ß-D-Glucan | 6/7 (86%) | 12/12 (100%) | 0.400 |
| *ß-D-Glucan* values *(pg/mL), median (IQR), n=17** | 430 (204-500) | 500 (342-2,594) | 0.300 |
| Positive mannan antigen | 2/3 (67%) | 9/12 (75%) | > 0.900 |
| Positive PCR | 2/2 (100%) | 14/18 (78%) | > 0.900 |
| Any CSF abnormality | 31/34 (91%) | 264/274 (96%) | 0.200 |
| Leukocytes > 5 cells | 28/35 (80%) | 212/232 (91%) | 0.063 |
| *Leucocytes (cels/mm³), median (IQR), n=253** | 95 (19-405) | 190 (40-658) | 0.074 |
| Lymphocytic predominance | 9/16 (56%) | 55/132 (42%) | 0.300 |
| *Lymphocytic (%), median (IQR), n=125** | 47 (36-80) | 31 (12-67) | 0.200 |
| Neutrophil predominance | 9/16 (56%) | 75/131 (57%) | > 0.900 |
| *Neutrophil (%), median (IQR), n=124** | 53 (18-64) | 56 (18-80) | 0.700 |
| Protein > 50 mg/dl | 28/29 (97%) | 202/225 (90%) | 0.300 |
| *Protein (mg/dl), median (IQR), n=246** | 200 (126-248) | 135 (80-294) | 0.500 |
| Glucose < 40 mg/dl | 23/28 (82%) | 112/202 (55%) | **0.007** |
| *Glucose (mg/dl), median (IQR), n=219** | 32 (25-39) | 34 (21-54) | 0.200 |
| **Positive histology** | 0/4 (0%) | 115/127 (91%) | **< 0.001** |

IQR: Interquartile range;

* Number of cases with available information among all patients for quantitative variables.

**Table S2.** Comparison between survivor and non-survivor groups of patients with central nervous system candidiasis for additional variables

| **Characteristics**  n = 403 | **Survivor**  n = 260 | **Non-survivor**  n = 143 | **p-value** |
| --- | --- | --- | --- |
| **Newborn characteristics** |  |  |  |
| Weight median, g (IQR), n=84* | 1.350 (988 - 1.760) | 1.050 (777 - 1.910) | 0.2 |
| Very low birth weight (<1500 g) | 44†/73§ (60%) | 14/21 (67%) | 0.6 |
| Preterm infant (GA <36 weeks) | 77/90 (86%) | 22/26 (85%) | >0.9 |
| Enlarged head circumference | 13/24 (54%) | 5/8 (62%) | >0.9 |
| ***Candida* species** |  |  |  |
| *Candida albicans* | 173 (68%) | 89 (63%) |  |
| *Candida auris* | 2 (0.8%) | 0 (0%) |  |
| *Candida dubliniensis* | 8 (3.1%) | 3 (2.1%) |  |
| *Candida famata* | 1 (0.4%) | 1 (0.7%) |  |
| *Candida glabrata* | 13 (5.1%) | 4 (2.8%) |  |
| *Candida guilliermondii* | 1 (0.4%) | 0 (0%) |  |
| *Candida kefyr* | 0 (0%) | 1 (0.7%) |  |
| *Candida krusei* | 1 (0.4%) | 1 (0.7%) |  |
| *Candida lusitaniae* | 2 (0.8%) | 1 (0.7%) |  |
| *Candida parapsilosis* | 17 (6.7%) | 7 (5.0%) |  |
| *Candida tropicalis* | 18 (7.1%) | 12 (8.5%) |  |
| **CSF findings** |  |  |  |
| Leukocyte count > 5 cells/μl | 163/181 (90%) | 67/74 (91%) | >0.9 |
| Leukocyte count, median, n=241* | 200 (50 - 670) | 108 (26 - 500) | 0.2 |
| Lymphocytic pleocytosis | 39/96 (41%) | 18/42 (43%) | 0.8 |
| Lymphocytic %, median, n=116* | 40 (15 - 66) | 35 (15 - 80) | >0.9 |
| Neutrophilic pleocytosis | 55/95 (58%) | 26/42 (62%) | 0.7 |
| Neutrophilic %, median, n=115* | 56 (27 - 76) | 62 (15 - 80) | >0.9 |
| Protein > 50 mg/dL | 153/171 (89%) | 66/71 (93%) | 0.4 |
| Protein, median, n=234* | 149 (80 - 300) | 156 (83 - 259) | 0.8 |
| Glucose < 40 mg/dL | 96/157 (61%) | 32/61 (52%) | 0.2 |
| Glucose, median, n=207* | 32 (20, 45) | 40 (24, 77) | 0.004 |
| Abscess drainage | 30/233 (13%) | 18/118 (15%) | 0.5 |
| **Site of infection** |  |  |  |
| Disseminated disease | 109/242 (45%) | 58/116 (50%) | 0.4 |
| **Treatment** |  |  |  |
| Ventricular shunt | 60/233 (26%) | 28/118 (24%) | 0.7 |
| Maximum dose of amphotericin B median, mg/kg/day (IQR), n=120* | 1.00 (1.00 - 3.00) | 1.00 (0.70 - 1.00) | 0.10 |
| Amphotericin deoxycholate | 100/224 (45%) | 32/97 (33%) | 0.051 |
| Sequelae | 34/194 (18%) | 2/15 (13%) | 0.7 |
| **Radiological findings** |  |  |  |
| No alterations | 14/101 (14%) | 4/33 (12%) | 0.8 |
| Leptomeningitis | 17/22 (77%) | 7/8 (88%) | >0.9 |
| Pachymeningitis | 5/22 (23%) | 2/8 (25%) | >0.9 |
| Abscess | 39/49 (80%) | 6/10 (60%) | 0.2 |
| Nodular | 17/49 (35%) | 4/10 (40%) | 0.7 |
| Multiple | 30/49 (61%) | 4/10 (40%) | 0.2 |
| Empyema | 4/99 (4%) | 1/31 (3%) | >0.9 |
| Haemorrhage | 5/10 (50%) | 5/7 (71%) | 0.6 |
| Ischemia | 7/10 (70%) | 6/7 (86%) | 0.6 |
| Bilateral | 4/10 (40%) | 3/7 (43%) | >0.9 |
| Deep | 2/10 (20%) | 3/7 (43%) | 0.6 |
| Aneurysm | 2/10 (20%) | 3/7 (43%) | 0.6 |
| **Outcomes** |  |  |  |
| Sequelae | 34/194 (18%) | 2/15 (13%) | 0.7 |
| IQR: Interquartile range; GA: Gestational age; CNS: Central nervous system; CSF: cerebrospinal fluid.  * Number of cases with available information among all patients for quantitative variables.  † Number of events with the specified characteristic for qualitative variables.  § Number of patients in each group with available information for qualitative variables. | | | |

**Table S3.** Multiple imputation vs. complete case

Ratio_OR_CC_MI is the complete-case OR divided by the imputed OR (1.00 = identical); Same_direction indicates whether both estimates fall on the same side of OR = 1.

**Table S4.** Characteristics of central nervous system candidiasis diagnoses according to diagnostic period

| **Characteristics** | **1945-1980**  **n=64** | **1981-2000**  **n=154** | **2001-2024**  **n=200** | **p-value** |
| --- | --- | --- | --- | --- |
| **Demographic characteristics** |  |  |  |  |
| Age median, years (IQR), n=416* | 30 (2-50) | 3 (0-32) | 26 (0-53) | <0.001 |
| Children | 22†/64§ (34%) | 88/152 (58%) | 80/200 (40%) | <0.001 |
| Newborn | 8/24 (33%) | 35/92 (38%) | 35/79 (44%) | 0.5 |
| Male | 38/64 (59%) | 78/144 (54%) | 137/199 (69%) | 0.019 |
| **Risk factors** |  |  |  |  |
| Immunosuppression | 5/64 (7.8%) | 32/154 (21%) | 40/192 (21%) | 0.050 |
| Previous neurosurgery | 2/64 (3.1%) | 35/149 (23%) | 55/200 (28%) | <0.001 |
| Abdominal surgery | 13/64 (20%) | 6/149 (4.0%) | 15/200 (7.5%) | <0.001 |
| Intravascular catheter use | 16/59 (27%) | 43/109 (39%) | 40/151 (26%) | 0.064 |
| Total parenteral nutrition | 3/42 (7.1%) | 28/106 (26%) | 20/145 (14%) | 0.006 |
| **Clinical characteristics** |  |  |  |  |
| Duration of symptoms median, weeks (IQR), n=197* | 2 (1-8) | 2 (1-5) | 3 (1-8) | 0.7 |
| Isolated CNS | 17/45 (38%) | 81/144 (56%) | 109/189 (58%) | 0.049 |
| Disseminated disease | 28/45 (62%) | 63/139 (45%) | 80/189 (42%) | 0.055 |
| **Diagnosis characteristics** |  |  |  |  |
| Definition |  |  |  | <0.001 |
| *Probable* | 1/64 (1.6%) | 6/154 (3.9%) | 44/200 (22%) |  |
| *Proven* | 63/64 (98%) | 148/154 (96%) | 156/200 (78%) |  |
| *Candida* species |  |  |  |  |
| *Candida albicans* | 50/64 (78%) | 105/148 (71%) | 116/198 (59%) |  |
| *Candida auris* | 0/64  (0%) | 0/148  (0%) | 3/198 (1.5%) |  |
| *Candida dubliniensis* | 0/64  (0%) | 0/148  (0%) | 11/198 (5.6%) |  |
| *Candida famata* | 0/64  (0%) | 0/148  (0%) | 2/198 (1.0%) |  |
| *Candida glabrata* | 0/64  (0%) | 6/148 (4.1%) | 11/198 (5.6%) |  |
| *Candida guillermondii* | 0/64  (0%) | 1/148 (0.7%) | 0/198  (0%) |  |
| *Candida kefyr* | 0/64  (0%) | 0/148  (0%) | 1/198 (0.5%) |  |
| *Candida krusei* | 0/64  (0%) | 0/148  (0%) | 2/198 (1.0%) |  |
| *Candida lusitaniae* | 0/64  (0%) | 3/148 (2.0%) | 0/198  (0%) |  |
| *Candida parapsilosis* | 0/64  (0%) | 4/148 (2.7%) | 20/198 (10%) |  |
| *Candida tropicalis* | 4/64 (6.2%) | 12/148 (8.1%) | 15/198 (7.6%) |  |
| Positive blood cultures | 13/31 (42%) | 44/100 (44%) | 59/130 (45%) | >0.9 |
| Cerebrospinal fluid |  |  |  |  |
| *Positive direct microscopy* | 10/15 (67%) | 24/53 (45%) | 38/71 (54%) | 0.3 |
| *Positive culture* | 24/47 (51%) | 111/128 (87%) | 116/164 (71%) | <0.001 |
| *Positive ß-D-Glucan* | 0/0 (NA%) | 0/0 (NA%) | 18/19 (95%) | >0.9 |
| Positive histology | 42/48 (88%) | 30/35 (86%) | 43/48 (90%) | >0.9 |
| Autopsy | 34/62 (55%) | 22/121 (18%) | 24/173 (14%) | <0.001 |
| *Diagnosis by autopsy* | 14/64 (22%) | 10/139 (7.2%) | 13/192 (6.8%) | <0.001 |
| **Treatment** |  |  |  |  |
| Neurosurgical treatment | 18/46 (39%) | 50/140 (36%) | 78/190 (41%) | 0.6 |
| Antifungal therapy | 22/45 (49%) | 127/140 (91%) | 186/193 (96%) | <0.001 |
| *Amphotericin B* | 18/36 (50%) | 111/139 (80%) | 124/189 (66%) | <0.001 |
| *Deoxycholate* | 18/39 (46%) | 85/117 (73%) | 30/169 (18%) | <0.001 |
| *Lipossomal* | 0/64 (0%) | 4/117 (3.4%) | 68/166 (41%) | <0.001 |
| *Echinocandin* | 0/36 (0%) | 0/137 (0%) | 27/189 (14%) | <0.001 |
| *Fluconazole* | 0/36 (0%) | 28/137 (20%) | 126/189 (67%) | <0.001 |
| *Voriconazole* | 0/36 (0%) | 0/137 (0%) | 44/189 (23%) | <0.001 |
| *Flucytosine* | 9/36 (25%) | 77/137 (56%) | 56/189 (30%) | <0.001 |
| *Combination therapy* | 7/36 (19%) | 77/137 (56%) | 87/189 (46%) | <0.001 |
| *Amphotericin B plus flucytosine* | 6/34 (18%) | 65/133 (49%) | 45/182 (25%) | <0.001 |
| *Amphotericin B plus fluconazole* | 0/34 (0%) | 3/133 (2.3%) | 24/183 (13%) | <0.001 |
| *Intrathecal amphotericin B* | 1/64 (1.6%) | 8/149 (5.4%) | 11/200 (5.5%) | 0.5 |
| **Outcomes** |  |  |  |  |
| Follow-up, median (months), n=239* | 6 (2, 16) | 4 (2, 12) | 3 (1, 12) | 0.3 |
| Death | 49/63 (78%) | 49/147 (33%) | 45/193 (23%) | <0.001 |

IQR: Interquartile range; CNS: Central nervous system.

* Number of cases with available information among all patients for quantitative variables.

† Number of events with the specified characteristic for qualitative variables.

§ Number of patients in each group with available information for qualitative variables.

**Table S5.** Risk of bias

A. Case reports

| **CHARACTERISTICS** | | | | **JOANNA BRIGGS INSTITUTE (JBI) CHECKLIST (Y=Yes; N=No; U=Unclear)** | | | | | | | |
| --- | --- | --- | --- | --- | --- | --- | --- | --- | --- | --- | --- |
| **Article** | **Year** | **Language** | **Design** | **1** | **2** | **3** | **4** | **5** | **6** | **7** | **8** |
| Martin | 2019 | English | Case report | Y | Y | Y | Y | Y | Y | Y | Y |
| Caria | 2022 | English | Case report | Y | Y | Y | Y | Y | Y | U | Y |
| Hiruma | 1986 | English | Case report | Y | Y | Y | Y | Y | Y | Y | Y |
| Crause | 2015 | English | Case report | Y | Y | Y | Y | Y | Y | Y | Y |
| Letournel | 2010 | English | Case report | Y | Y | Y | Y | Y | Y | Y | Y |
| Stevenson | 1961 | English | Case report | Y | Y | Y | Y | Y | Y | Y | Y |
| Finelli | 2018 | English | Case report | U | Y | Y | Y | Y | Y | Y | Y |
| Bruinsma-Adams | 1991 | English | Case report | Y | Y | Y | Y | Y | Y | Y | Y |
| Burnie | 1986 | English | Case report | Y | Y | Y | Y | Y | Y | Y | Y |
| Wu | 2022 | English | Case report | Y | U | Y | Y | Y | U | U | Y |
| Shankar | 2016 | English | Case report | Y | U | Y | Y | Y | Y | U | Y |
| Radhouanea | 2020 | English | Case report | Y | U | Y | Y | Y | Y | Y | Y |
| Hong | 2007 | English | Case report | Y | U | Y | Y | Y | Y | Y | Y |
| Neves | 2014 | English | Case report | Y | Y | Y | Y | Y | Y | U | Y |
| Kamitsuka | 1995 | English | Case report | Y | Y | Y | Y | Y | Y | Y | Y |
| Borha | 2008 | French | Case report | Y | Y | Y | Y | Y | Y | Y | Y |
| Soto-Hernández | 2000 | English | Case report | Y | Y | Y | Y | Y | Y | U | Y |
| Rienzo | 2014 | English | Case report | Y | Y | Y | Y | Y | Y | Y | Y |
| Aleixo | 2000 | English | Case report | Y | Y | Y | Y | Y | Y | U | Y |
| Jarløv | 1995 | English | Case report | Y | Y | Y | Y | Y | Y | Y | Y |
| Ludviksson | 1993 | English | Case report | Y | Y | Y | Y | Y | Y | Y | Y |
| Bourbeau | 2021 | English | Case report | Y | Y | Y | Y | Y | Y | U | Y |
| Ahmadi | 2009 | English | Case report | Y | U | Y | Y | Y | Y | U | Y |
| Lilien | 1978 | English | Case report | Y | Y | Y | Y | Y | Y | Y | Y |
| Roe | 1972 | English | Case report | Y | Y | Y | Y | Y | Y | Y | Y |
| Taneja | 2009 | English | Case report | U | U | Y | Y | Y | Y | Y | Y |
| Pihet | 2005 | French | Case report | Y | Y | Y | Y | Y | Y | U | Y |
| Lopez | 2020 | English | Case report | Y | Y | Y | Y | Y | Y | Y | Y |
| Chen | 2021 | English | Case report | Y | U | Y | Y | Y | Y | Y | Y |
| Fennelly | 2013 | English | Case report | Y | U | Y | Y | Y | Y | U | Y |
| Sánchez-Portocarrero | 1994 | English | Case report | Y | Y | Y | Y | Y | Y | U | Y |
| Price | 2023 | English | Case report | Y | U | Y | Y | Y | Y | Y | Y |
| van Hal | 2008 | English | Case report | Y | Y | Y | Y | Y | Y | Y | Y |
| Shapira | 1974 | English | Case report | Y | Y | Y | Y | Y | Y | Y | Y |
| Prinsloo | 2003 | English | Case report | U | U | Y | Y | Y | Y | U | Y |
| Winters | 1995 | English | Case report | Y | Y | Y | Y | U | Y | U | Y |
| Colomba | 2014 | English | Case report | Y | Y | Y | Y | Y | Y | U | Y |
| Burgert | 1994 | English | Case report | Y | Y | Y | Y | Y | Y | Y | Y |
| Bisharat | 1996 | English | Case report | Y | Y | Y | Y | Y | Y | Y | Y |
| Marr | 1994 | English | Case report | Y | Y | Y | Y | Y | Y | Y | Y |
| Sarmal | 1993 | English | Case report | Y | U | Y | Y | Y | Y | U | Y |
| Sanchez | 1987 | English | Case report | Y | Y | Y | Y | Y | Y | Y | Y |
| DeVita | 1966 | English | Case report | Y | Y | Y | Y | Y | Y | Y | Y |
| Tissot | 2002 | French | Case report | Y | Y | Y | Y | Y | Y | Y | Y |
| Mercer | 1978 | English | Case report | Y | Y | Y | Y | Y | Y | Y | Y |
| Porrillo | 1962 | English | Case report | N | Y | Y | Y | Y | Y | Y | Y |
| Farrugia | 2016 | English | Case report | Y | Y | Y | Y | Y | Y | Y | Y |
| Yuan | 2021 | English | Case report | Y | Y | Y | Y | Y | Y | Y | Y |
| Sanya | 2006 | English | Case report | Y | Y | Y | Y | Y | Y | U | Y |
| Smego | 1984 | English | Case report | Y | Y | Y | Y | Y | Y | Y | Y |
| Brenier-Pinchart | 1999 | English | Case report | Y | Y | Y | Y | Y | Y | U | Y |
| Fadel H. | 2018 | English | Case report | Y | Y | Y | Y | Y | Y | Y | Y |
| O'brian | 2010 | English | Case report | U | Y | Y | Y | Y | Y | Y | Y |
| Farshad | 2009 | English | Case report | Y | Y | Y | Y | Y | Y | Y | Y |
| Sidrim | 2011 | English | Case report | Y | Y | Y | Y | Y | Y | Y | Y |
| Corti | 2011 | English | Case report | Y | Y | Y | Y | Y | Y | Y | Y |
| Roger | 1983 | English | Case report | Y | Y | Y | Y | Y | Y | Y | Y |
| Kim | 2022 | English | Case report | Y | Y | Y | Y | Y | Y | Y | Y |
| Goldani | 2010 | English | Case report | Y | Y | Y | Y | Y | Y | Y | Y |
| Yoganathan | 2014 | English | Case report | Y | Y | Y | Y | Y | Y | Y | Y |
| Hirnle | 2023 | English | Case report | Y | Y | Y | Y | Y | Y | Y | Y |
| Sanjeev | 2003 | English | Case report | Y | Y | Y | Y | Y | Y | Y | Y |
| Kuruoglu | 2019 | Turkish | Case report | Y | Y | Y | Y | Y | Y | Y | Y |
| Cao | 2021 | English | Case report | Y | Y | Y | Y | Y | Y | Y | Y |
| Tahir | 2020 | English | Case report | Y | Y | Y | Y | Y | Y | U | Y |
| Kuenzli | 2023 | English | Case report | Y | Y | Y | Y | Y | Y | Y | Y |
| Shen | 2023 | English | Case report | Y | Y | Y | Y | Y | Y | Y | Y |
| Airas | 2009 | English | Case report | Y | Y | Y | Y | Y | Y | Y | Y |
| Goldsmith | 1990 | English | Case report | Y | Y | Y | Y | Y | Y | Y | Y |
| Baradkar | 2008 | English | Case report | Y | Y | Y | Y | Y | Y | U | Y |
| Black | 1970 | English | Case report | Y | Y | Y | Y | Y | Y | Y | Y |
| Thron | 1981 | English | Case report | Y | Y | Y | Y | Y | Y | U | Y |
| Zhang | 2014 | English | Case report | Y | Y | Y | Y | Y | Y | Y | Y |
| Jorge | 2003 | Spanish | Case report | Y | Y | Y | Y | Y | Y | Y | Y |
| Tweddle | 1998 | English | Case report | Y | Y | Y | Y | Y | Y | Y | Y |
| Figueiredo | 2013 | English | Case report | Y | Y | Y | Y | Y | Y | U | Y |
| Ikeda | 1990 | English | Case report | Y | Y | Y | Y | Y | Y | Y | Y |
| Andreen | 2023 | English | Case report | Y | Y | Y | Y | Y | Y | Y | Y |
| Herbst | 2015 | English | Case report | Y | Y | Y | Y | Y | Y | Y | Y |
| Giotaki | 2022 | English | Case report | Y | Y | Y | Y | Y | Y | Y | Y |
| Herrera | 2019 | English | Case report | Y | Y | Y | Y | Y | Y | Y | Y |
| Voice | 1994 | English | Case report | Y | Y | Y | Y | Y | Y | Y | Y |
| Ralph | 1996 | English | Case report | Y | Y | Y | Y | Y | Y | Y | Y |
| Asif | 2021 | English | Case report | Y | Y | Y | Y | Y | Y | U | Y |
| Biesbroek | 2013 | English | Case report | Y | Y | Y | Y | Y | Y | Y | Y |
| Bhalla | 2018 | English | Case report | Y | Y | Y | Y | Y | Y | Y | Y |
| Ziwei | 2023 | Chinese | Case report | Y | Y | Y | Y | Y | Y | Y | Y |
| Raja | 2021 | English | Case report | Y | Y | Y | Y | N | Y | Y | N |
| Lai | 1997 | English | Case report | Y | Y | Y | Y | Y | Y | Y | Y |
| Strickland | 2018 | English | Case report | Y | Y | Y | Y | Y | Y | Y | Y |
| Bismay | 2012 | English | Case report | Y | Y | Y | Y | Y | Y | U | Y |
| Morris | 1945 | English | Case report | Y | Y | Y | Y | Y | Y | Y | Y |
| Prabhu | 2004 | English | Case report | Y | Y | Y | Y | Y | Y | Y | Y |
| Epelbaum | 1993 | English | Case report | Y | Y | Y | Y | Y | Y | Y | Y |
| Gheshlaghi | 2019 | English | Case report | Y | Y | Y | Y | Y | Y | U | Y |
| Berkeley | 2008 | English | Case report | Y | Y | Y | Y | Y | Y | Y | Y |
| Jans | 2013 | English | Case report | Y | Y | Y | Y | Y | Y | Y | Y |
| Gurses | 1996 | English | Case report | Y | Y | Y | Y | Y | Y | Y | Y |
| Bassiri-Jahromi | 2014 | English | Case report | Y | Y | Y | Y | Y | Y | U | Y |
| Decker | 2018 | English | Case report | Y | Y | Y | Y | Y | Y | Y | Y |
| Yampolsky | 2010 | English | Case report | Y | Y | Y | Y | Y | Y | U | Y |
| Miri | 2012 | English | Case report | Y | Y | Y | Y | Y | Y | Y | Y |
| Eschwege | 1957 | English | Case report | Y | Y | Y | Y | Y | Y | Y | Y |
| Berenguer | 1996 | English | Case report | Y | Y | Y | Y | Y | Y | Y | Y |
| Peterson | 1992 | English | Case report | Y | Y | Y | Y | U | Y | U | Y |
| Chin | 2021 | English | Case report | Y | Y | Y | Y | Y | Y | Y | Y |
| Marazzi | 2007 | English | Case report | Y | Y | Y | Y | Y | Y | Y | Y |
| Britt | 1981 | English | Case report | Y | Y | Y | Y | Y | Y | Y | Y |
| Kaji | 1998 | English | Case report | Y | Y | Y | Y | Y | Y | N | Y |
| Lucasti | 2023 | English | Case report | Y | Y | Y | Y | Y | Y | Y | Y |
| Argersinger | 2021 | English | Case report | Y | Y | Y | Y | Y | Y | Y | Y |
| Merwick | 2015 | English | Case report | Y | Y | Y | Y | Y | Y | Y | Y |
| Kozlowski | 2015 | English | Case report | Y | Y | Y | Y | Y | Y | Y | Y |
| Koch | 2003 | English | Case report | Y | N | Y | Y | Y | Y | U | Y |
| Liberti | 1975 | English | Case report | Y | Y | Y | Y | Y | Y | Y | Y |
| Choudhari | 2007 | English | Case report | Y | Y | Y | Y | Y | Y | Y | Y |
| Santangelo | 2022 | English | Case report | Y | Y | Y | Y | Y | Y | Y | Y |
| Alfred | 1995 | English | Case report | Y | Y | Y | Y | Y | Y | Y | Y |
| Tang | 2022 | English | Case report | Y | Y | Y | Y | Y | Y | U | Y |
| Baradkar | 2007 | English | Case report | Y | Y | Y | Y | Y | U | U | Y |
| Yamahiro | 2016 | English | Case report | Y | Y | Y | Y | Y | Y | Y | Y |
| Chadwick | 1980 | English | Case report | Y | Y | Y | Y | Y | Y | Y | Y |
| Duclos | 2014 | Francês | Case report | Y | Y | Y | Y | Y | Y | Y | Y |
| Wittig | 1973 | Português | Case report | Y | Y | Y | Y | Y | Y | Y | Y |
| Emdin | 1954 | English | Case report | Y | Y | Y | Y | Y | Y | Y | Y |
| Zhu | 2017 | English | Case report | Y | Y | Y | Y | Y | Y | Y | Y |
| Van Cutsem | 1986 | English | Case report | Y | Y | Y | Y | Y | Y | Y | Y |
| Erduran | 1998 | English | Case report | Y | Y | Y | Y | Y | Y | Y | Y |
| Geiger | 1946 | English | Case report | Y | Y | Y | Y | Y | Y | Y | Y |
| Wee | 2021 | English | Case report | Y | Y | Y | Y | Y | Y | Y | Y |
| Singh | 2006 | English | Case report | Y | N | Y | Y | N | N | N | Y |
| Barone | 1995 | English | Case report | Y | Y | Y | Y | Y | Y | Y | Y |
| Carter | 2008 | English | Case report | Y | Y | Y | Y | Y | Y | Y | Y |
| Cevit | 1999 | English | Case report | Y | Y | Y | Y | Y | Y | Y | Y |
| Wong | 2006 | English | Case report | Y | Y | Y | Y | Y | Y | Y | Y |
| de Mol | 1982 | English | Case report | Y | Y | Y | Y | Y | Y | Y | Y |
| Lin | 2013 | English | Case report | Y | Y | Y | Y | Y | Y | Y | Y |
| Iwashita | 1978 | English | Case report | Y | Y | Y | Y | Y | U | U | Y |
| Maschke | 1999 | English | Case report | N | N | N | Y | N | Y | N | Y |
| Jeantin | 2022 | English | Case report | Y | Y | Y | Y | Y | Y | Y | Y |
| Kuzdan | 2014 | English | Case report | Y | Y | Y | Y | Y | Y | Y | Y |
| Sanjuan | 2023 | English | Case report | Y | Y | Y | Y | Y | Y | Y | Y |
| Lin | 2009 | English | Case report | Y | Y | Y | Y | Y | Y | Y | Y |
| Miranda | 2021 | English | Case report | Y | Y | Y | Y | Y | Y | Y | Y |
| Mason | 1995 | English | Case report | Y | Y | Y | Y | Y | Y | Y | Y |
| Leggiadro | 1988 | English | Case report | Y | Y | Y | Y | Y | Y | U | Y |
| DeJonge | 1997 | English | Case report | Y | Y | Y | Y | Y | Y | Y | Y |
| McAbee | 1995 | English | Case report | Y | Y | Y | Y | Y | Y | Y | Y |
| Guo | 2014 | English | Case report | Y | Y | Y | Y | Y | Y | U | Y |
| Bozynski | 1986 | English | Case report | Y | Y | Y | Y | Y | Y | Y | Y |
| Dawson | 2004 | English | Case report | Y | Y | Y | Y | Y | Y | Y | Y |
| Turhan | 2009 | English | Case report | Y | Y | Y | Y | Y | Y | Y | Y |
| Liu | 2004 | English | Case report | Y | Y | Y | Y | Y | Y | Y | Y |
| Iao | 2015 | English | Case report | Y | Y | Y | Y | Y | Y | Y | Y |
| Auriti | 2016 | English | Case report | Y | Y | Y | Y | Y | Y | U | Y |
| Mirhendi | 2022 | English | Case report | Y | Y | Y | Y | Y | Y | U | Y |
| Ho | 1982 | English | Case report | Y | Y | Y | Y | Y | Y | Y | Y |
| Raffa | 2021 | English | Case report | Y | Y | Y | Y | Y | U | U | Y |
| Sakayama | 2002 | English | Case report | Y | Y | Y | Y | Y | Y | Y | Y |
| Celmeli | 2016 | English | Case report | Y | Y | Y | Y | Y | Y | Y | Y |
| Behre | 2008 | English | Case report | Y | Y | Y | Y | Y | Y | Y | Y |
| Singhal | 2018 | English | Case report | Y | Y | Y | Y | Y | Y | Y | Y |
| Phanthumchinda | 1991 | English | Case report | Y | Y | Y | Y | Y | Y | Y | Y |
| Esposito | 2005 | French | Case report | Y | Y | Y | Y | Y | Y | Y | Y |
| Tonami | 1989 | English | Case report | Y | Y | Y | Y | Y | Y | Y | Y |
| Andrew | 2011 | English | Case report | Y | Y | Y | Y | Y | Y | U | Y |
| Morison | 1988 | English | Case report | Y | Y | Y | Y | Y | Y | Y | Y |
| Mendes | 2008 | English | Case report | Y | Y | Y | Y | Y | Y | Y | Y |
| Kirpekar | 1986 | English | Case report | U | Y | Y | Y | U | U | U | Y |
| Hurlbert | 1995 | English | Case report | Y | Y | Y | Y | Y | Y | Y | Y |
| Cao | 2014 | English | Case report | Y | Y | Y | Y | Y | Y | Y | Y |
| Lisch | 1994 | German | Case report | Y | Y | Y | Y | Y | Y | Y | Y |
| Cruciani | 1992 | English | Case report | Y | Y | Y | Y | Y | Y | Y | Y |
| Grimes | 1998 | English | Case report | Y | Y | Y | Y | Y | Y | Y | Y |
| Jamjoom | 1992 | English | Case report | Y | Y | Y | Y | Y | Y | Y | Y |
| Tsakiri | 2018 | English | Case report | Y | Y | U | Y | Y | Y | Y | Y |
| Ceccarelli | 2016 | English | Case report | Y | Y | Y | Y | Y | Y | Y | Y |
| Nesrin Guzlen | 2007 | English | Case report | Y | Y | Y | Y | Y | Y | Y | Y |
| Nordstrom | 1977 | English | Case report | Y | Y | Y | Y | Y | Y | Y | Y |
| Gorell J M | 1979 | English | Case report | Y | Y | Y | Y | N | Y | Y | Y |
| Chattopadhyay | 1981 | English | Case report | Y | Y | Y | Y | Y | Y | Y | Y |
| Henig | 1967 | English | Case report | Y | Y | Y | Y | Y | Y | U | Y |
| Sarmiento | 1994 | English | Case report | Y | Y | Y | Y | Y | Y | Y | Y |
| Beeching/Benjamin | 2013 | English | Case report | U | Y | Y | Y | U | Y | U | Y |
| Moran | 2018 | English | Case report | Y | Y | Y | Y | Y | Y | Y | Y |
| Askari | 2024 | English | Case report | Y | Y | Y | Y | Y | Y | Y | Y |
| Hoshide | 2013 | English | Case report | Y | Y | Y | Y | Y | U | Y | Y |
| Kantor | 1984 | English | Case report | Y | Y | Y | Y | Y | Y | Y | Y |
| Anhalt | 1986 | English | Case report | Y | Y | Y | Y | Y | Y | Y | Y |
| Ozsváth | 2011 | English | Case report | U | U | U | Y | Y | Y | Y | Y |
| Rao | 1979 | English | Case report | Y | Y | Y | Y | Y | Y | Y | Y |
| Jinxiao | 2021 | Chinese | Case report | Y | Y | Y | Y | Y | Y | Y | Y |
| White | 1971 | English | Case report | Y | N | Y | Y | Y | Y | U | Y |
| Maximova | 2012 | English | Case report | Y | Y | Y | Y | U | Y | Y | Y |
| Binning | 2009 | English | Case report | Y | Y | Y | Y | Y | Y | Y | Y |
| Holyst | 1976 | English | Case report | Y | Y | Y | Y | Y | Y | Y | Y |
| Zimmerman | 1947 | English | Case report | Y | Y | Y | Y | Y | Y | Y | Y |
| Guerreiro | 2006 | English | Case report | Y | Y | Y | Y | Y | Y | Y | Y |
| Halpert | 1946 | English | Case report | Y | Y | Y | Y | N | Y | Y | Y |
| Xu | 2011 | English | Case report | Y | Y | Y | Y | Y | Y | Y | Y |
| Adachi | 2018 | English | Case report | Y | Y | Y | Y | N | Y | Y | Y |
| Vinh | 2014 | English | Case report | Y | U | Y | Y | U | Y | U | Y |
| Goudjil | 2016 | English | Case report | Y | Y | Y | Y | Y | Y | U | Y |
| Maggiori | 1982 | English | Case report | Y | Y | Y | Y | Y | Y | Y | Y |
| Ehni | 1987 | English | Case report | Y | Y | Y | Y | Y | Y | Y | Y |
| Kumar | 1979 | English | Case report | Y | Y | Y | Y | Y | Y | U | Y |
| Lenoir | 1989 | English | Case report | Y | Y | Y | Y | Y | Y | Y | Y |
| Ponomarev | 2023 | Russian | Case report | Y | Y | Y | Y | N | Y | Y | Y |
| Bashall | 2023 | English | Case report | Y | Y | Y | Y | Y | Y | U | Y |
| Fine | 1955 | English | Case report | Y | Y | Y | Y | Y | Y | Y | Y |
| Rocha | 2021 | English | Case report | Y | Y | Y | Y | Y | Y | U | Y |
| Ferrari | 2023 | English | Case report | Y | N | Y | Y | Y | U | U | Y |
| Lobato | 2023 | English | Case report | Y | Y | Y | Y | Y | Y | Y | Y |

B. **Case series**

| **CHARACTERISTICS** | | | | **JOANNA BRIGGS INSTITUTE (JBI)  CHECKLIST (Y=Yes; N=No; U=Unclear)** | | | | | | | | | |
| --- | --- | --- | --- | --- | --- | --- | --- | --- | --- | --- | --- | --- | --- |
| **Article** | **Year** | **Language** | **Design** | **1** | **2** | **3** | **4** | **5** | **6** | **7** | **8** | **9** | **10** |
| Salvatore | 2015 | English | Case series | Y | Y | Y | U | U | Y | Y | Y | Y | Y |
| O’Brien | 2011 | English | Case series | Y | Y | Y | Y | Y | Y | Y | Y | Y | Y |
| Casado | 1998 | English | Case series | Y | Y | Y | Y | Y | Y | Y | Y | Y | Y |
| Rodríguez-Arrondo | 1998 | English | Case series | Y | Y | Y | U | U | Y | Y | Y | Y | Y |
| Bridges | 2017 | English | Case series | Y | Y | Y | Y | Y | Y | Y | Y | Y | Y |
| Aldress | 2013 | English | Case series | N | Y | Y | U | U | Y | Y | Y | Y | Y |
| Bayer | 1976 | English | Case series | Y | Y | Y | Y | Y | Y | Y | Y | Y | Y |
| Kelly | 2022 | English | Case series | Y | Y | Y | Y | Y | Y | Y | Y | Y | Y |
| Livramento | 1989 | Portuguese | Case series | Y | Y | Y | U | U | Y | Y | N | Y | N |
| Zhu | 2021 | English | Case series | Y | Y | Y | Y | U | Y | Y | Y | Y | Y |
| Chen | 2004 | English | Case series | Y | Y | Y | Y | Y | Y | Y | Y | Y | Y |
| Geers | 1999 | English | Case series | Y | Y | Y | Y | N | Y | Y | Y | Y | Y |
| Smego | 1984 | English | Case series | Y | Y | Y | Y | Y | Y | Y | Y | Y | Y |
| Lunel | 2004 | English | Case series | N | Y | Y | U | U | Y | Y | Y | Y | Y |
| Chiou | 1994 | English | Case series | Y | Y | Y | Y | Y | Y | Y | Y | Y | Y |
| Lanternier | 2015 | English | Case series | Y | Y | Y | U | U | Y | Y | Y | Y | Y |
| Elias | 2009 | English | Case series | Y | Y | Y | Y | Y | Y | Y | Y | Y | Y |
| Nguyen | 1995 | English | Case series | Y | Y | Y | Y | Y | Y | Y | Y | Y | Y |
| Mao | 2012 | English | Case series | Y | Y | Y | Y | U | Y | Y | Y | Y | Y |
| Vorreith | 1968 | English | Case series | Y | Y | Y | Y | Y | Y | Y | Y | Y | Y |
| Chen | 1993 | English | Case series | Y | Y | Y | Y | Y | Y | Y | Y | Y | Y |
| Pahud | 2009 | English | Case series | N | Y | Y | U | U | Y | Y | Y | Y | Y |
| Huttova | 1998 | English | Case series | Y | Y | Y | Y | Y | Y | Y | Y | Y | Y |
| Queiroz | 1976 | Portuguese | Case series | Y | Y | Y | Y | Y | Y | Y | Y | Y | Y |
| Xu | 2021 | English | Case series | Y | Y | Y | Y | Y | Y | Y | Y | Y | Y |
| Faix | 1984 | English | Case series | Y | Y | Y | Y | Y | Y | Y | Y | Y | Y |
| Parker | 1977 | English | Case series | Y | Y | Y | Y | Y | Y | Y | Y | Y | Y |
| Murphy | 2000 | English | Case series | N | Y | Y | U | U | Y | Y | Y | Y | Y |
| Mahboubi | 1976 | English | Case series | N | Y | Y | U | U | Y | Y | Y | Y | Y |
| Medeiros | 2000 | English | Case series | Y | Y | Y | Y | Y | Y | Y | Y | Y | Y |
| Huang | 1998 | English | Case series | Y | Y | Y | Y | Y | Y | Y | Y | Y | Y |
| Scott | 1998 | English | Case series | N | Y | Y | U | U | Y | Y | Y | Y | Y |
| Toprak | 2015 | English | Case series | Y | Y | Y | Y | Y | Y | Y | Y | Y | Y |
| Mori | 1992 | English | Case series | Y | U | U | N | N | Y | Y | Y | N | Y |
| Chesney | 1976 | English | Case series | N | Y | Y | U | U | Y | Y | Y | Y | Y |
| Casado | 1997 | English | Case series | Y | Y | Y | Y | Y | Y | Y | Y | Y | Y |

**JBI Checklist**

**Case reports**

1. Were patient’s demographic characteristics clearly described?

2. Was the patient’s history clearly described and presented as a timeline?

3. Was the current clinical condition of the patient on presentation clearly described?

4. Were diagnostic tests or methods and the results clearly described?

5. Was the intervention(s) or treatment procedure(s) clearly described?

6. Was the post-intervention clinical condition clearly described?

7. Were adverse events (harms) or unanticipated events identified and described?

8. Does the case report provide takeaway lessons?

**Case series**

1. Were there clear criteria for inclusion in the case series?

2. Was the condition measured in a standard, reliable way for all participants included in the case series?

3. Were valid methods used for identification of the condition for all participants included in the case series?

4. Did the case series have consecutive inclusion of participants?

5. Did the case series have complete inclusion of participants?

6. Was there clear reporting of the demographics of the participants in the study?

7. Was there clear reporting of clinical information of the participants?

8. Were the outcomes or follow-up results of cases clearly reported?

9. Was there clear reporting of the presenting site(s)/clinic(s) demographic information?

10. Was statistical analysis appropriate?

**Appendix 1.** Search strategy

**Pubmed / Medline**
Date: July 4th, 2024
No date of publication or Language restriction

(“Candida” OR “Candidal” OR “Candidiasis” OR "Candida infection" OR "Candida infections" OR "infection, Candida" OR "disseminated candidiasis" OR "invasive candidiasis" OR "Candida spp." OR "Candida albicans" OR "Candida tropicalis" OR "Candida parapsilosis" OR "Candida glabrata" OR "Candida krusei" OR "Candida guilliermondii") AND (“central nervous system” OR “neurological involvement” OR “meningitis” OR “meningitides” OR “pachymeningitis” OR “leptomeningitis” OR “encephalitis” OR “meningoencephalitis” OR “brain inflammation” OR “ventriculitis” OR “hydrocephalus” OR “vasculitis” OR “stroke” OR “brain infarction” OR “intracerebral hemorrhage” OR “myelitis” OR “myelopathy” OR “spinal cord inflammation” OR “brain” OR “spinal cord”)
Records: 2.632 results

**Embase**

Date: July 4th, 2024
No date of publication or Language restriction

(Candida OR Candidal OR Candidiasis OR 'Candida infection' OR 'Candida infections' OR 'infection, Candida' OR 'disseminated candidiasis' OR 'invasive candidiasis' OR 'Candida spp.' OR 'Candida albicans' OR 'Candida tropicalis' OR 'Candida parapsilosis' OR 'Candida glabrata' OR 'Candida krusei' OR 'Candida guilliermondii' ) AND ('central nervous system' OR 'neurological involvement' OR meningitis OR meningitides OR pachymeningitis OR leptomeningitis OR encephalitis OR meningoencephalitis OR 'brain inflammation' OR ventriculitis OR hydrocephalus OR vasculitis OR stroke OR 'brain infarction' OR 'intracerebral hemorrhage' OR myelitis OR myelopathy OR 'spinal cord inflammation' OR brain OR 'spinal cord' )

Records: 3.943

**Appendix 2.** Missing data and multiple imputation

The proportion of missing data for every variable considered for the multivariable mortality model ranged from 0.0% to 27.3%. The figure and table below illustrates the number of missing values for each variable included in the multivariable model.

The outcome (mortality) was missing in 15 patients (3.6%). Only 226 of 418 patients (54.1%) had all model variables simultaneously observed. Neuroimaging and β-D-glucan results were missing for 67% and 95% of patients, respectively. Because this cohort comprises case reports and case series published over approximately 80 years, we considered this missingness to be structural and not at random (MNAR), as the absence of a documented result most likely reflects that the test was unavailable or not performed at the time and setting of publication, rather than a normal or negative finding. Accordingly, neuroimaging and β-D-glucan variables were excluded from the imputed multivariable model. The variables that are only defined or fully observed after the outcome occurs (e.g., follow-up, clinical/radiological outcome at last follow-up, recurrence) were excluded a priori from the predictor set, as they are partly determined by survival itself and would introduce reverse-causation/immortal-time bias.

Candidate predictors were selected based on clinical relevance and univariable screening (decade of publication, age, sex, haematological malignancy, immunosuppressive drugs, altered mental status, meningeal irritation signs, positive CSF culture, combination antifungal therapy, and previous neurosurgery); age was modelled as a linear term and decade of publication was included as a proxy for changes in diagnostic and therapeutic practice over time. The model provided 14.3 events per parameter (143 events / 10 parameters), and the maximum variance-inflation factor across imputed datasets was 1.67, indicating no problematic collinearity.

Missing predictor values were handled by Multiple Imputation by Chained Equations (MICE, R package 'mice'). The imputation method was chosen according to the measurement scale of each variable: predictive mean matching for continuous variables and logistic regression for binary variables. The imputation model included exactly the variables of the substantive model plus the outcome; no auxiliary variables were used. Mortality was not imputed; therefore, patients with unknown vital status (n = 15) were excluded from the multivariable analysis. The number of imputed datasets (m = 46) was set to at least the percentage of incomplete cases, with 20 iterations per chain. Convergence was assessed from trace plots of the mean and standard deviation of the imputed values across iterations and chains, as shown below.

Plausibility of the imputations was assessed separately by variable type: kernel density plots comparing observed and imputed values for continuous variables, and comparison of the observed versus imputed category distributions for categorical variables, as shown below. The estimates from the m logistic regression models were combined using Rubin's rules; the mean fraction of missing information across model terms was 0.11.

**Appendix 3.** Additional references

1.	[Salvatore, C. M. *et al.* (1→3)-β-d-Glucan in Cerebrospinal Fluid as a Biomarker for Candida and Aspergillus Infections of the Central Nervous System in Pediatric Patients. *J Pediatric Infect Dis Soc* **5**, 277–286 (2016).](http://paperpile.com/b/W31M7G/Bl5n)

2.	[Martin, S. *et al.* A 7-Year-Old Child With Headaches and Prolonged Fever Associated With Oral and Nail Lesions. *Open Forum Infect Dis* **6**, ofz229 (2019).](http://paperpile.com/b/W31M7G/P67f)

3.	[Caria, J. *et al.* A case of central nervous system infection by in an immunosuppressed patient with HIV-1 infection. *Med Mycol Case Rep* **38**, 21–24 (2022).](http://paperpile.com/b/W31M7G/VaJR)

4.	[Hiruma, M., Nitta, M. & Kagawa, S. A case of cerebrospinal fluid shunt colonization and obstruction by Candida albicans. *Mykosen* **29**, 455–460 (1986).](http://paperpile.com/b/W31M7G/hQCB)

5.	[Stevenson, J. & Kennedy, A. C. A fatal case of agranulocytosis due to thenalidine tartrate complicated by acute renal failure and mycelial abscesses of brain. *Scott. Med. J.* **6**, 522–525 (1961).](http://paperpile.com/b/W31M7G/pi18)

6.	[Finelli, P. F. A fatal case of undiagnosed candida meningitis: Role of computer-assisted diagnosis. *Neurologist* **23**, 138–140 (2018).](http://paperpile.com/b/W31M7G/EYj4)

7.	[Bruinsma-Adams, I. K. AIDS presenting as Candida albicans meningitis: a case report. *AIDS* **5**, 1268–1269 (1991).](http://paperpile.com/b/W31M7G/hzpA)

8.	[Burnie, J. P., Matthews, R. C., Watson, J. G., Milligan, D. W. & Tabaqchali, S. Analysis of the antibody response developing in an infant with Candida albicans meningitis. *J. Infect.* **12**, 79–83 (1986).](http://paperpile.com/b/W31M7G/WABy)

9.	[Wu, B., Xian Lee, S., Suter, K. & Villanueva, D.-D. An unusual presentation of CNS candidiasis. *Chest* **162**, A958 (2022).](http://paperpile.com/b/W31M7G/Yu52)

10.	[Shankar, S. *et al.* A rare case of candida brain abscess secondary to metastatic tongue cancer on initial presentation. *Chest* **150**, 448A (2016).](http://paperpile.com/b/W31M7G/eMjD)

11.	[Radhouane, K. *et al.* Brain abscess due to Candida glabrata in an immunocompetent patient. A case report with update and literature review. *IDCases* **22**, e00996 (2020).](http://paperpile.com/b/W31M7G/1Syo)

12.	[Hong, X.-Y., Chou, Y.-C. & Lazareff, J. A. Brain stem candidiasis mimicking cerebellopontine angle tumor. *Surg. Neurol.* **70**, 87–91 (2008).](http://paperpile.com/b/W31M7G/6CNo)

13.	[Neves, N., Santos, L., Reis, C. & Sarmento, A. Candida albicans brain abscesses in an injection drug user patient: a case report. *BMC Res. Notes* **7**, 837 (2014).](http://paperpile.com/b/W31M7G/9Qfd)

14.	[Kamitsuka, M. D., Nugent, N. A., Conrad, P. D. & Swanson, T. N. Candida albicans brain abscesses in a premature infant treated with amphotericin B, flucytosine and fluconazole. *Pediatr. Infect. Dis. J.* **14**, 329–331 (1995).](http://paperpile.com/b/W31M7G/vDk1)

15.	[Borha, A. *et al.* Granulome cérébral à Candida albicans chez un patient immunocompétent. Cas clinique. *Neurochirurgie* **55**, 57–62 (2009).](http://paperpile.com/b/W31M7G/32LW)

16.	[Soto-Hernández, J. L., Ramírez-Crescencio, M. A., Moreno Estrada, V. M. & del Valle Robles, R. Candida albicans cerebral granulomas associated with a nonfunctional cerebrospinal fluid shunt: case report. *Neurosurgery* **47**, 973–6; discussion 976–7 (2000).](http://paperpile.com/b/W31M7G/ku6D)

17.	[Di Rienzo, A. *et al.* Candida albicans Dural granuloma: Case report. *NMC Case Rep. J.* **2**, 61–64 (2015).](http://paperpile.com/b/W31M7G/O5ne)

18.	[Aleixo, M. J., Caldeira, L. & Ferreira, M. L. Candida albicans meningitis: Clinical case. *J. Infect.* **40**, 191–192 (2000).](http://paperpile.com/b/W31M7G/a6jo)

19.	[Jarløv, J. O., Born, P. & Bruun, B. Candida albicans meningitis in a 27 weeks premature infant treated with liposomal amphotericin-B (AmBisome). *Scand. J. Infect. Dis.* **27**, 419–420 (1995).](http://paperpile.com/b/W31M7G/mO9C)

20.	[Ludviksson, B. R., Thorarensen, O., Gudnason, T. & Halldorsson, S. Candida albicans meningitis in a child with myeloperoxidase deficiency. *Pediatr. Infect. Dis. J.* **12**, 162–164 (1993).](http://paperpile.com/b/W31M7G/1ly7)

21.	[Bourbeau, K., Gupta, S. & Wang, S. Candida albicans meningitis in AIDS patient: A case report and literature review. *IDCases* **25**, e01216 (2021).](http://paperpile.com/b/W31M7G/oDod)

22.	[Ahmadi, F. *et al.* Candida albicans meningitis in an infant with Noonan syndrome. *Braz. J. Infect. Dis.* **13**, 452–453 (2009).](http://paperpile.com/b/W31M7G/nReg)

23.	[Lilien, L. D., Ramamurthy, R. S. & Pildes, R. S. Candida albicans meningitis in a premature neonate successfully treated with 5-fluorocytosine and amphotericin B: a case report and review of the literature. *Pediatrics* **61**, 57–61 (1978).](http://paperpile.com/b/W31M7G/usIa)

24.	[Roe, D. C. Candida albicans meningitis successfully treated with amphotericin B. *Arch. Pediatr. Adolesc. Med.* **124**, 926 (1972).](http://paperpile.com/b/W31M7G/P4q5)

25.	[Taneja, J., Mishra, B., Thakur, A., Dogra, V. & Loomba, P. Candida albicans meningitis with ventriculitis in a four-year-old child. *Neurol. India* **57**, 682–684 (2009).](http://paperpile.com/b/W31M7G/0bXQ)

26.	[Pihet, M., Poulain, D., De Sèze, J., Camus, D. & Sendid, B. Candida albicans meningo-encephalo-myelo-radiculitis at an addict. *Ann. Biol. Clin. (Paris)* **63**, 547–552 (2005).](http://paperpile.com/b/W31M7G/WSUd)

27.	[Lopez, A. A., Kazina, C., Del Bigio, M. R. & Fanella, S. Candida albicans ventriculoperitoneal shunt infection in an infant: A case report and review of the literature. *Off. J. Assoc. Med. Microbiol. Infect. Dis. Can.* **5**, 35–38 (2020).](http://paperpile.com/b/W31M7G/Vcco)

28.	[Chen, H. *et al.* Candida central nervous system infection after neurosurgery: a single-institution case series and literature review. *Ann. Palliat. Med.* **10**, 11362–11369 (2021).](http://paperpile.com/b/W31M7G/vqIf)

29.	[Fennelly, A. M., Slenker, A. K., Murphy, L. C., Moussouttas, M. & DeSimone, J. A. Candida cerebral abscesses: a case report and review of the literature. *Med. Mycol.* **51**, 779–784 (2013).](http://paperpile.com/b/W31M7G/knlO)

30.	[Sánchez-Portocarrero, J., Martín-Rabadán, P., Saldaña, C. J. & Pérez-Cecilia, E. Candida cerebrospinal fluid shunt infection. Report of two new cases and review of the literature. *Diagn. Microbiol. Infect. Dis.* **20**, 33–40 (1994).](http://paperpile.com/b/W31M7G/GXZ5)

31.	[Price, C., Wilson, I. & Catchpoole, E. Candida dubliniensis chronic meningitis in an immunocompetent patient: Case report and literature review. *IDCases* **31**, e01665 (2023).](http://paperpile.com/b/W31M7G/MAAW)

32.	[van Hal, S. J., Stark, D., Harkness, J. & Marriott, D. Candida dubliniensis meningitis as delayed sequela of treated C. dubliniensis fungemia. *Emerg. Infect. Dis.* **14**, 327–329 (2008).](http://paperpile.com/b/W31M7G/gzFv)

33.	[Shapira, Y., Drucker, M., Russell, A. & Yarom, R. Candida endocarditis and encephalitis in an infant. *Clin. Pediatr. (Phila.)* **13**, 542–546 (1974).](http://paperpile.com/b/W31M7G/v4q6)

34.	[Prinsloo, B., Weldhagen, G. F. & Blaine, R. W. Candida famata central nervous system infection. *S. Afr. Med. J.* **93**, 601–602 (2003).](http://paperpile.com/b/W31M7G/kmzy)

35.	[Winters, W. D., Shaw, D. W. & Weinberger, E. Candida fungus balls presenting as intraventricular masses in cranial sonography. *J. Clin. Ultrasound* **23**, 266–270 (1995).](http://paperpile.com/b/W31M7G/ZCOO)

36.	[Colomba, C. *et al.* Candida glabrata meningitis and endocarditis: a late severe complication of candidemia. *Int. J. Infect. Dis.* **29**, 174–175 (2014).](http://paperpile.com/b/W31M7G/TXAs)

37.	[O’Brien, D. *et al.* Candida infection of the central nervous system following neurosurgery: a 12-year review. *Acta Neurochir. (Wien)* **153**, 1347–1350 (2011).](http://paperpile.com/b/W31M7G/Cpg8)

38.	[Burgert, S. J., Classen, D. C., Burke, J. P. & Blatter, D. D. Candidal brain abscess associated with vascular invasion: a devastating complication of vascular catheter-related candidemia. *Clin. Infect. Dis.* **21**, 202–205 (1995).](http://paperpile.com/b/W31M7G/27VF)

39.	[Bisharat, N., Reichman, N., Luboshitzky, R., Raz, R. & Flatau, E. Candidal meningitis in a patient with chronic mucocutaneous candidiasis. *Infect. Dis. Clin. Pract. (Baltim. Md.)* **5**, 138 (1996).](http://paperpile.com/b/W31M7G/Ibmf)

40.	[Casado, J. L., Quereda, C. & Corral, I. Candidal meningitis in HIV-infected patients. *AIDS Patient Care STDS* **12**, 681–686 (1998).](http://paperpile.com/b/W31M7G/cEut)

41.	[Casado, J. L. *et al.* Candidal meningitis in HIV-infected patients: analysis of 14 cases. *Clin. Infect. Dis.* **25**, 673–676 (1997).](http://paperpile.com/b/W31M7G/OMOp)

42.	[Rodríguez-Arrondo, F. *et al.* Candidal meningitis in HIV-infected patients: treatment with fluconazole. *Scand. J. Infect. Dis.* **30**, 417–418 (1998).](http://paperpile.com/b/W31M7G/SDVk)

43.	[Marr, B., Gross, S., Cunningham, C. & Weiner, L. Candidal sepsis and meningitis in a very-low-birth-weight infant successfully treated with fluconazole and flucytosine. *Clin. Infect. Dis.* **19**, 795–796 (1994).](http://paperpile.com/b/W31M7G/qAvX)

44.	[Sarma, P. S., Durairaj, P. & Padhye, A. A. Candida lusitaniae causing fatal meningitis. *Postgrad. Med. J.* **69**, 878–880 (1993).](http://paperpile.com/b/W31M7G/jWlR)

45.	[Sanchez, P. J. & Cooper, B. H. Candida lusitaniae: sepsis and meningitis in a neonate. *Pediatr. Infect. Dis. J.* **6**, 758–759 (1987).](http://paperpile.com/b/W31M7G/Nq1r)

46.	[DeVita, V. T., 2nd, Utz, J. P., Williams, T. & Carbone, P. P. Candida meningitis. *Arch. Intern. Med.* **117**, 527–535 (1966).](http://paperpile.com/b/W31M7G/HwX4)

47.	[Tissot, B. & Bramary, F. Méningite à Candida après ponction–biopsie hépatique. *Rev. Med. Interne* **23**, 477–478 (2002).](http://paperpile.com/b/W31M7G/zxSN)

48.	[Bridges, K. J., Li, R., Fleseriu, M. & Cetas, J. S. Candida meningitis after transsphenoidal surgery: A single-institution case series and literature review. *World Neurosurg.* **108**, 41–49 (2017).](http://paperpile.com/b/W31M7G/MqWE)

49.	[Mercer, H. P. & Gupta, J. M. Candida meningitis causing aqueductal stenosis following parenteral nutrition in an infant with meconium peritonitis. *Aust. Paediatr. J.* **14**, 286–287 (1978).](http://paperpile.com/b/W31M7G/bIa2)

50.	[Parrillo, O. J., Meiberger, M. & Elston, H. Candida meningitis complicating Hodgkin’s disease. Apparent recovery with amphotericin B therapy. *JAMA* **182**, 189–191 (1962).](http://paperpile.com/b/W31M7G/4psR)

51.	[Farrugia, M. K. *et al.* Candida meningitis in an immunocompetent patient detected through (1→3)-beta-d-glucan. *Int. J. Infect. Dis.* **51**, 25–26 (2016).](http://paperpile.com/b/W31M7G/Cp0t)

52.	[Yuan, L. *et al.* Candida meningitis in an infant after abdominal surgery successfully treated with intrathecal and intravenous amphotericin B: A case report. *Medicine (Baltimore)* **100**, e27205 (2021).](http://paperpile.com/b/W31M7G/M63i)

53.	[Sanya, E. O., Ameen, N. B. & Onile, B. A. Candida meningitis in a suspected immunosuppressive patient--a case report. *West Afr. J. Med.* **25**, 79–81 (2006).](http://paperpile.com/b/W31M7G/tIQU)

54.	[Aldress, K., Al Shaalan, M., Memish, Z., Alola, S. & Bannatyne, R. Candida meningitis in children: report of two cases. *J. Chemother.* **12**, 339–344 (2000).](http://paperpile.com/b/W31M7G/FjfW)

55.	[Smego, R. A., Jr *et al.* Candida meningitis in two children with severe combined immunodeficiency. *J. Pediatr.* **104**, 902–904 (1984).](http://paperpile.com/b/W31M7G/Rg9u)

56.	[Brenier-Pinchart, M. P., Leclercq, P., Mallié, M. & Bettega, G. Candida meningitis possibly resulting from a harpoon injury. *Eur. J. Clin. Microbiol. Infect. Dis.* **18**, 454–455 (1999).](http://paperpile.com/b/W31M7G/fsNC)

57.	[Bayer, A. S., Edwards, J. E., Jr, Seidel, J. S. & Guze, L. B. Candida meningitis. Report of seven cases and review of the english literature. *Medicine (Baltimore)* **55**, 477–486 (1976).](http://paperpile.com/b/W31M7G/GPhp)

58.	[Kelly, L. *et al.* meningitis/ventriculitis over a decade. Increased morbidity and length of stay a concern. *Br J Neurosurg* **37**, 227–230 (2023).](http://paperpile.com/b/W31M7G/ainZ)

59.	[Fadel, H. *et al.* Candida parapsilosis infection of ventriculoperitoneal shunt in adult: Case report and literature review. *World Neurosurg.* **119**, 290–293 (2018).](http://paperpile.com/b/W31M7G/ERg3)

60.	[O’Brien, D. *et al.* Candida parapsilosis meningitis associated with Gliadel (BCNU) wafer implants. *Br. J. Neurosurg.* **25**, 289–291 (2011).](http://paperpile.com/b/W31M7G/8BKh)

61.	[Bagheri, F., Cervellione, K. L., Maruf, M., Marino, W. & Santucci, T., Jr. Candida parapsilosis meningitis associated with shunt infection in an adult male. *Clin. Neurol. Neurosurg.* **112**, 248–251 (2010).](http://paperpile.com/b/W31M7G/2ax2)

62.	[Sidrim, J. J. C. *et al.* Candida parapsilosis meningitis as the first manifestation of AIDS: case report. *J. Med. Microbiol.* **60**, 1530–1533 (2011).](http://paperpile.com/b/W31M7G/Maxa)

63.	[Corti, M. *et al.* Candida parapsilosis meningitis in a patient with AIDS. Report of a case and review of the literature. *Rev. Iberoam. Micol.* **30**, 122–124 (2013).](http://paperpile.com/b/W31M7G/WNdv)

64.	[Faix, R. G. Candida parapsilosis meningitis in a premature infant. *Pediatr. Infect. Dis.* **2**, 462–464 (1983).](http://paperpile.com/b/W31M7G/VXEj)

65.	[Kim, J. J. *et al.* Candida shunt infection causing arachnoiditis and hydrocephalus: A case report. *Cureus* **14**, e23675 (2022).](http://paperpile.com/b/W31M7G/EBKB)

66.	[Goldani, L. Z. & Santos, R. P. Candida tropicalis as an emerging pathogen in Candida meningitis: case report and review. *Braz. J. Infect. Dis.* **14**, 631–633 (2010).](http://paperpile.com/b/W31M7G/Jolr)

67.	[Yoganathan, S. *et al.* Candida tropicalis brain abscess in a neonate: An emerging nosocomial menace. *Ann. Indian Acad. Neurol.* **17**, 448–450 (2014).](http://paperpile.com/b/W31M7G/HvfN)

68.	[Hirnle, G., Kapałka, M., Krawiec, M. & Hrapkowicz, T. Candida tropicalis endocarditis on the aortic valve with coexisting meningitis in a patient with multiple risk factors - What to do? *IDCases* **36**, e01949 (2024).](http://paperpile.com/b/W31M7G/jmiw)

69.	[Ahuja, S. R., Karande, S., Kulkarni, M. V. & Tendolkar, U. Candida tropicalis meningitis in a young infant. *Indian J. Pediatr.* **70**, 925–927 (2003).](http://paperpile.com/b/W31M7G/2Ibb)

70.	[Kuruoğlu, T., Çelik, M., Çelmeli, F. & Tanyel, E. CARD9 mutation in a patient with Candida albicans meningoencephalitis; A case report. *Mikrobiyol. Bul.* **55**, 656–664 (2021).](http://paperpile.com/b/W31M7G/3JgR)

71.	[Cao, X.-G., Yu, C.-W., Zhou, S.-S., Huang, Y. & Wang, C.-Y. Case report: A Candida meningitis in an immunocompetent patient detected through the next-generation sequencing. *Front. Med. (Lausanne)* **8**, 656066 (2021).](http://paperpile.com/b/W31M7G/RYj2)

72.	[Tahir, M., Peseski, A. M. & Jordan, S. J. Case Report: Candida dubliniensis as a Cause of Chronic Meningitis. *Front. Neurol.* **11**, 601242 (2020).](http://paperpile.com/b/W31M7G/EZ8V)

73.	[Kuenzli, A. B. *et al.* Case report: Chronic Candida albicans meningitis: a rare entity diagnosed by metagenomic next-generation sequencing. *Front. Cell. Infect. Microbiol.* **14**, 1322847 (2024).](http://paperpile.com/b/W31M7G/ZSdW)

74.	[Shen, H. *et al.* Case report: Successful treatment of recurrent Candida albicans meningitis with Kimura’s disease using amphotericin B colloidal dispersion combined with fluconazole. *Infect. Drug Resist.* **16**, 6905–6909 (2023).](http://paperpile.com/b/W31M7G/MYLU)

75.	[Airas, L. *et al.* Central nervous system immune reconstitution inflammatory syndrome (IRIS) after hematopoietic SCT. *Bone Marrow Transplant.* **45**, 593–596 (2010).](http://paperpile.com/b/W31M7G/ePq5)

76.	[Goldsmith, L. S., Rubenstein, S. D., Wolfson, B. J., Faerber, E. N. & Fisher, M. C. Cerebral calcifications in a neonate with candidiasis. *Pediatr. Infect. Dis. J.* **9**, 451–453 (1990).](http://paperpile.com/b/W31M7G/yfKs)

77.	[Baradkar, V. P., Mathur, M. & Kumar, S. Cerebral Candida abscess in an infant. *Indian J. Med. Microbiol.* **27**, 70–72 (2009).](http://paperpile.com/b/W31M7G/92E8)

78.	[Black, J. T. Cerebral candidiasis: case report of brain abscess secondary to Candida albicans, and review of literature. *J. Neurol. Neurosurg. Psychiatry* **33**, 864–870 (1970).](http://paperpile.com/b/W31M7G/dP33)

79.	[Thron, A. & Wiethölter, H. Cerebral candidiasis: CT studies in a case of brain abscess and granuloma due to Candida albicans. *Neuroradiology* **23**, 223–225 (1982).](http://paperpile.com/b/W31M7G/9qmt)

80.	[Zhang, S.-C. Cerebral candidiasis in a 4-year-old boy after intestinal surgery. *J. Child Neurol.* **30**, 391–393 (2015).](http://paperpile.com/b/W31M7G/C1lb)

81.	[Mata Jorge, M., Pino Vázquez, A., Santos García, J. G., Oyágüez Ugidos, P. & Aragón García, M^a^. Candidiasis cerebral en un recién nacido. *An. Pediatr. (Barc.)* **58**, 194–194 (2003).](http://paperpile.com/b/W31M7G/OegB)

82.	[Tweddle, D. A., Graham, J. C., Shankland, G. S. & Kernahan, J. Cerebral candidiasis in a child 1 year after leukaemia. *Br. J. Haematol.* **103**, 795–797 (1998).](http://paperpile.com/b/W31M7G/qXiM)

83.	[Figueiredo, S. M. *et al.* Cerebral macroabscess caused by Candida albicans in an immunocompetent patient: A diagnostic challenge. *Med. Mycol. Case Rep.* **3**, 17–19 (2014).](http://paperpile.com/b/W31M7G/Sxx1)

84.	[Ikeda, K., Yamashita, J., Fujisawa, H. & Fujita, S. Cerebral granuloma and meningitis caused by Candida albicans: useful monitoring of mannan antigen in cerebrospinal fluid. *Neurosurgery* **26**, 860–863 (1990).](http://paperpile.com/b/W31M7G/nDPc)

85.	[Livramento, J. A., Machado, L. R., Moreno-Carvalho, O. A., Lacaz, C. S. & Spina-França, A. Líquido cefalorraqueano em infecção do sistema nervoso central por leveduras do gênero Candida: análise de 11 casos. *Arq. Neuropsiquiatr.* **47**, 332–336 (1989).](http://paperpile.com/b/W31M7G/DWss)

86.	[Andreen, N., Brink, M., Hammarström, H. & Yilmaz, A. Chronic Candida albicans meningitis following critical COVID-19 and serial measurements of (1,3)-β-D-glucan in cerebrospinal fluid. *Infect. Dis. (Lond.)* **55**, 794–797 (2023).](http://paperpile.com/b/W31M7G/muR9)

87.	[Herbst, M. *et al.* Chronic candida albicans meningitis in a 4-year-old girl with a homozygous mutation in the CARD9 gene (Q295X). *Pediatr. Infect. Dis. J.* **34**, 999–1002 (2015).](http://paperpile.com/b/W31M7G/a5OP)

88.	[Giotaki, I. *et al.* Chronic Candida albicans meningitis misdiagnosed as polymyalgia rheumatica and successfully treated with voriconazole. *Clin. Case Rep.* **10**, e05664 (2022).](http://paperpile.com/b/W31M7G/foAi)

89.	[Herrera, S. *et al.* Chronic Candida dubliniensis meningitis in a lung transplant recipient. *Med. Mycol. Case Rep.* **24**, 41–43 (2019).](http://paperpile.com/b/W31M7G/QJYK)

90.	[Voice, R. A., Bradley, S. F., Sangeorzan, J. A. & Kauffman, C. A. Chronic candidal meningitis: an uncommon manifestation of candidiasis. *Clin. Infect. Dis.* **19**, 60–66 (1994).](http://paperpile.com/b/W31M7G/0w1y)

91.	[Ralph, E. D. & Hussain, Z. Chronic meningitis caused by Candida albicans in a liver transplant recipient: usefulness of the polymerase chain reaction for diagnosis and for monitoring treatment. *Clin. Infect. Dis.* **23**, 191–192 (1996).](http://paperpile.com/b/W31M7G/KFaz)

92.	Asif, A. [*et al*](http://paperpile.com/b/W31M7G/oRwq). [Widespread Embolic Strokes Involving the Bilateral Supra- and Infratentorial Brain Territory: A Unique Radiological Manifestation of Candida Endocarditis.](http://paperpile.com/b/W31M7G/aVNE) Annals of Neurology (2021).

93.	[Zhu, Y., Gong, X., Li, Z., Wang, D. & Yan, C. Clinical analysis of intravenous and oral sequential treatment with voriconazole for Candida central nervous system infection in six premature infants. *Front. Pharmacol.* **12**, 631293 (2021).](http://paperpile.com/b/W31M7G/Iowt)

94.	[Chen, T.-L. *et al.* Clinical characteristics, treatment and prognostic factors of candidal meningitis in a teaching hospital in Taiwan. *Scand. J. Infect. Dis.* **36**, 124–130 (2004).](http://paperpile.com/b/W31M7G/oRwq)

95.	[Geers, T. A. & Gordon, S. M. Clinical significance of Candida species isolated from cerebrospinal fluid following neurosurgery. *Clin. Infect. Dis.* **28**, 1139–1147 (1999).](http://paperpile.com/b/W31M7G/j52U)

96.	[Smego, R. A., Perfect, J. R. & Durack, D. T. Combined therapy with amphotericin B and 5-fluorocytosine for candida meningitis. *Clin. Infect. Dis.* **6**, 791–801 (1984).](http://paperpile.com/b/W31M7G/ONmW)

97.	[Biesbroek, J. M., Verduyn Lunel, F. M., Kragt, J. J., Amelink, G. J. & Frijns, C. J. M. Culture-negative Candida meningitis diagnosed by detection of Candida mannan antigen in CSF. *Neurology* **81**, 1555–1556 (2013).](http://paperpile.com/b/W31M7G/QHzB)

98.	[Verduyn Lunel, F. M. *et al.* Detection of the *Candida* antigen Mannan in Cerebrospinal fluid specimens from patients suspected of having *Candida* meningitis. *J. Clin. Microbiol.* **42**, 867–870 (2004).](http://paperpile.com/b/W31M7G/Bza1)

99.	[Bhalla, G. S. *et al.* Device-associated central nervous system infection caused by Candida parapsilosis. *Cureus* **10**, e3140 (2018).](http://paperpile.com/b/W31M7G/EJTa)

100.	[Tao Z. W., Shen Y. L. & Weng Y. L. A case report of Candida albicans meningitis. *Zhonghua Nei Ke Za Zhi* **63**, 97–99 (2024).](http://paperpile.com/b/W31M7G/K1G6)

101.	[Lai, P. H., Lin, S. M., Pan, H. B. & Yang, C. F. Disseminated miliary cerebral candidiasis. *AJNR Am. J. Neuroradiol.* **18**, 1303–1306 (1997).](http://paperpile.com/b/W31M7G/VPhq)

102.	[Strickland, B. A. *et al.* Endoscopic endonasal transsphenoidal drainage of a spontaneous Candida glabrata pituitary abscess. *World Neurosurg.* **109**, 467–470 (2018).](http://paperpile.com/b/W31M7G/0OKP)

103.	[Bismay, K. *et al.* Disseminated candidiasis 18 years after renal transplantation. *Indian J. Nephrol.* **22**, 462–465 (2012).](http://paperpile.com/b/W31M7G/hAQ2)

104.	[Morris, A. A., Kalz, G. G. & Lotspeich, E. S. Ependymitis and meningitis due to Candida (Monilia) albicans. *Arch. Neurol. Psychiatry* **54**, 361–366 (1945).](http://paperpile.com/b/W31M7G/5STS)

105.	[Prabhu, R. M. & Orenstein, R. Failure of caspofungin to treat brain abscesses secondary to Candida albicans prosthetic valve endocarditis. *Clin. Infect. Dis.* **39**, 1253–1254 (2004).](http://paperpile.com/b/W31M7G/uppe)

106.	[Epelbaum, S., Laurent, C., Morin, G., Berquin, P. & Piussan, C. Failure of fluconazole treatment in Candida meningitis. *J. Pediatr.* **123**, 168–169 (1993).](http://paperpile.com/b/W31M7G/50cH)

107.	[Gheshlaghi, M. & Helweg-Larsen, J. Fatal chronic meningitis caused by Candida dubliniensis after liver transplantation. *Med. Mycol. Case Rep.* **27**, 22–24 (2020).](http://paperpile.com/b/W31M7G/Iom0)

108.	[Berkeley, J. L., Nath, A. & Pardo, C. A. Fatal immune reconstitution inflammatory syndrome with human immunodeficiency virus infection and Candida meningitis: case report and review of the literature. *J. Neurovirol.* **14**, 267–276 (2008).](http://paperpile.com/b/W31M7G/X1hN)

109.	[Jans, J., Brüggemann, R. J. M., Christmann, V., Verweij, P. E. & Warris, A. Favorable outcome of neonatal cerebrospinal fluid shunt-associated Candida meningitis with caspofungin. *Antimicrob. Agents Chemother.* **57**, 2391–2393 (2013).](http://paperpile.com/b/W31M7G/08eu)

110.	[Gürses, N. & Kalayci, A. G. Fluconazole monotherapy for candidal meningitis in a premature infant. *Clin. Infect. Dis.* **23**, 645–646 (1996).](http://paperpile.com/b/W31M7G/3FWW)

111.	[Bassiri-Jahromi, S. & Iravani, K. Fungal brain abscess: report of three cases and review of literature. *Asian Pac. J. Trop. Dis.* **4**, S854–S859 (2014).](http://paperpile.com/b/W31M7G/TbM0)

112.	[Decker, B. M., Noyes, C. D., Ramundo, M. B. & Thomas, A. A. Fungal cauda equina lesion with delayed cord compression and treatment response. *Clin. Neurol. Neurosurg.* **174**, 185–186 (2018).](http://paperpile.com/b/W31M7G/WMvt)

113.	[Yampolsky, C., Corti, M. & Negroni, R. Fungal cerebral abscess in a diabetic patient successfully treated with surgery followed by prolonged antifungal therapy. *Rev. Iberoam. Micol.* **27**, 6–9 (2010).](http://paperpile.com/b/W31M7G/gBPW)

114.	[Miri, S. M., Tayebi Meybodi, A., Habibi, Z. & Mohseni, M. Fungal granuloma of the brain in a case of chronic mucocutaneous candidiasis. *Arch. Iran. Med.* **15**, 452–454 (2012).](http://paperpile.com/b/W31M7G/Qtj3)

115.	[Chiou, C. C. *et al.* Fungal infection of ventriculoperitoneal shunts in children. *Clin. Infect. Dis.* **19**, 1049–1053 (1994).](http://paperpile.com/b/W31M7G/QnxP)

116.	[Eschwege, J. Generalized moniliasis with localization in the brain. *AMA Arch. Neurol. Psychiatry* **79**, 250–263 (1958).](http://paperpile.com/b/W31M7G/3h0h)

117.	[Berenguer, J. *et al.* Genetic dissimilarity of two fluconazole-resistant Candida albicans strains causing meningitis and oral candidiasis in the same AIDS patient. *J. Clin. Microbiol.* **34**, 1542–1545 (1996).](http://paperpile.com/b/W31M7G/5XxT)

118.	[Peterson, P. K., Chao, C. C., Hu, S., Thielen, K. & Shaskan, E. G. Glioblastoma, transforming growth factor-beta, and Candida meningitis: a potential link. *Am. J. Med.* **92**, 262–264 (1992).](http://paperpile.com/b/W31M7G/akDj)

119.	[Chin, Y. H., Yap, Y. L., Chin, H. H. & Lau, B. K. Hypothalamic-pituitary fungal infection causing panhypopituitarism. *Med. J. Malaysia* **76**, 606–609 (2021).](http://paperpile.com/b/W31M7G/XZ2d)

120.	[Lanternier, F. *et al.* Inherited CARD9 deficiency in otherwise healthy children and adults with Candida species-induced meningoencephalitis, colitis, or both. *J. Allergy Clin. Immunol.* **135**, 1558–68.e2 (2015).](http://paperpile.com/b/W31M7G/hpHI)

121.	[Marazzi, M. G., Bondi, E., Giannattasio, A., Strozzi, M. & Savioli, C. Intracranial aneurysm associated with chronic mucocutaneous candidiasis. *Eur. J. Pediatr.* **167**, 461–463 (2008).](http://paperpile.com/b/W31M7G/5Fsq)

122.	[Britt, R. H., Enzmann, D. R. & Remington, J. S. Intracranial infection in cardiac transplant recipients. *Ann. Neurol.* **9**, 107–119 (1981).](http://paperpile.com/b/W31M7G/bmkI)

123.	[Kaji, M., Shoji, H. & Oizumi, K. Intractable meningitis and intracranial abscess following sinusitis due to Candida species. *Kurume Med. J.* **45**, 279–281 (1998).](http://paperpile.com/b/W31M7G/EcBJ)

124.	[Lucasti, C., Scott, M. M., Vallee, E. K. & Kowalski, J. Intradural Candida Albicans infection that presented as epidural abscess: A case report. *Int. J. Surg. Case Rep.* **107**, 108337 (2023).](http://paperpile.com/b/W31M7G/SFrh)

125.	[Argersinger, D. P. *et al.* Intradural cauda equina Candida abscess presenting with hydrocephalus: case report. *J. Neurosurg. Spine* **31**, 890–893 (2019).](http://paperpile.com/b/W31M7G/Cnc8)

126.	[Merwick, Á. *et al.* Intradural extramedullary spinal candida infection. *Pract. Neurol.* **15**, 400–404 (2015).](http://paperpile.com/b/W31M7G/Qk2x)

127.	[Kozlowski, E. A. Intraparenchymal hemorrhage and fungemia in a preterm neonate. *J. Diagn. Med. Sonogr.* **31**, 382–385 (2015).](http://paperpile.com/b/W31M7G/zaIv)

128.	[Elias, M. L. *et al.* Isolation of cryptococcus, Candida, Aspergillus, Rhodotorula and nocardia from meningitis patients in egypt. *J. Egypt. Public Health Assoc.* **84**, 169–181 (2009).](http://paperpile.com/b/W31M7G/jio2)

129.	[Koch, S., Rudel, B. & Tietz, H.-J. Lethal otogenic Candida meningitis. *Mycoses* **47**, 450–453 (2004).](http://paperpile.com/b/W31M7G/IO0T)

130.	[Di Liberti, J. H. & Hughes, R. A. Letter: Another case of disseminated candidiasis. *Pediatrics* **55**, 897–898 (1975).](http://paperpile.com/b/W31M7G/qNab)

131.	[Choudhari, Y. K. & Choudhari, K. A. Life-threatening fungal meningitis-ventriculitis secondary to vaginal candidiasis. *Br. J. Hosp. Med. (Lond.)* **68**, 274–275 (2007).](http://paperpile.com/b/W31M7G/2Cat)

132.	[Santangelo, G., Stone, J., Johnson, M. & Walter, K. Lumbar cistern Candida intradural abscess following epidural anesthesia. *Surg. Neurol. Int.* **13**, 435 (2022).](http://paperpile.com/b/W31M7G/Dhne)

133.	[Lindner, A. *et al.* Magnetic resonance image findings of spinal intramedullary abscess caused by Candida albicans: case report. *Neurosurgery* **36**, 411–412 (1995).](http://paperpile.com/b/W31M7G/Ac8Q)

134.	[Tang, X., Xu, Y. & Qiu, S. Magnetic resonance imaging features of tropicalis candidal meningoencephalitis. *JAMA Neurol.* **79**, 305–306 (2022).](http://paperpile.com/b/W31M7G/N2kX)

135.	[Baradkar, V. & Taklikar, S. Meningitis caused by Candida albicans in a premature neonate. *J. Pediatr. Neurosci.* **2**, 90 (2007).](http://paperpile.com/b/W31M7G/t4uQ)

136.	[Yamahiro, A., Lau, K. H. V., Peaper, D. R. & Villanueva, M. Meningitis caused by Candida dubliniensis in a patient with cirrhosis: A case report and review of the literature. *Mycopathologia* **181**, 589–593 (2016).](http://paperpile.com/b/W31M7G/StBC)

137.	[Nguyen, M. H. & Yu, V. L. Meningitis caused by Candida species: an emerging problem in neurosurgical patients. *Clin. Infect. Dis.* **21**, 323–327 (1995).](http://paperpile.com/b/W31M7G/mr3D)

138.	[Chadwick, D. W., Hartley, E. & Mackinnon, D. M. Meningitis caused by Candida tropicalis. *Arch. Neurol.* **37**, 175–176 (1980).](http://paperpile.com/b/W31M7G/vMNk)

139.	[Duclos, G., Dumont, J.-C., Ranque, S., Zieleskiewicz, L. & Bruder, N. Méningite à Candida albicans chez l’adulte, apport des nouveaux moyens diagnostiques. *Ann. Fr. Anesth. Reanim.* **33**, 533–535 (2014).](http://paperpile.com/b/W31M7G/bYNy)

140.	[Wittig, E. O., Lopes, R. W. & Kasting, G. Meningencefalite a cândida. *Arq. Neuropsiquiatr.* **31**, 146–150 (1973).](http://paperpile.com/b/W31M7G/ZPpe)

141.	[Mao, J. *et al.* MRI-DWI improves the early diagnosis of brain abscess induced by Candida albicans in preterm infants. *Transl. Pediatr.* **1**, 76–84 (2012).](http://paperpile.com/b/W31M7G/iLAQ)

142.	[Zhu, Z. *et al.* Multiple brain abscesses caused by infection with Candida glabrata: A case report." Experimental and therapeutic medicine. *Exp. Ther. Med.* **15**, 2374–2380 (2018).](http://paperpile.com/b/W31M7G/hfhC)

143.	[Van Cutsem, E., Boogaerts, M. A., Tricot, G. & Verwilghen, R. L. Multiple Brain Abscesses Caused by Torulopsis glabrata in an Immunocompromised Patient/Durch Torulopsis glabrata hervorgerufene Hirnabszesse bei einem immungeschwächten Patienten. *Mycoses* **29**, 306–308 (1986).](http://paperpile.com/b/W31M7G/Qp1l)

144.	[Erduran, E., Yildiran, A. & Gedik, Y. Multiple fungal brain abscess in a child with acute myeloblastic leukemia. *Pediatr. Hematol. Oncol.* **15**, 463–466 (1998).](http://paperpile.com/b/W31M7G/Fwev)

145.	[Vorreith, M. Mycotic encephalitis. *Acta Neuropathol.* **11**, 55–68 (1968).](http://paperpile.com/b/W31M7G/Ky6i)

146.	[Geiger, A. J. & Wenner, H. A. Mycotic endocarditis and meningitis; report of a case due to Monilia albicans. *Yale J. Biol. Med.* **18**, 259–268 (1946).](http://paperpile.com/b/W31M7G/UsyO)

147.	[Wee, L. E., Wong, C. S. L., Tan, A. L. & Oh, H. M.-L. Negative cerebrospinal fluid β-d-glucan levels as an indicator for treatment cessation ahead of biochemical resolution: A case report of Candida glabrata meningitis. *Med. Mycol. Case Rep.* **32**, 47–49 (2021).](http://paperpile.com/b/W31M7G/47Rz)

148.	[Singh, S., Chowdhury, V., Dixit, R. & Prakash, A. Neonatal candida infection of the CNS : A case report. *Indian J. Radiol. Imaging* **16**, 711 (2006).](http://paperpile.com/b/W31M7G/n1ar)

149.	[Barone, S. R. & Krilov, L. R. Neonatal candidal meningitis in a full-term infant with congenital cutaneous candidiasis. *Clin. Pediatr. (Phila.)* **34**, 217–219 (1995).](http://paperpile.com/b/W31M7G/PJmt)

150.	[Carter, J. E., Laurini, J. A., Evans, T. N. & Estrada, B. Neonatal Candida parapsilosis meningitis and empyema related to epidural migration of a central venous catheter. *Clin. Neurol. Neurosurg.* **110**, 614–618 (2008).](http://paperpile.com/b/W31M7G/hfs7)

151.	[Chen, J. Y. Neonatal candidiasis associated with meningitis and endophthalmitis. *Acta Paediatr. Jpn.* **36**, 261–265 (1994).](http://paperpile.com/b/W31M7G/0rHj)

152.	[Cevi̇t, Ö. *et al.* Neonatal cerebral candida abscess: Radiologic and clinical findings. *Turk. J. Med. Sci.* **29**, 345–348 (1999).](http://paperpile.com/b/W31M7G/8q47)

153.	[Wong, K. K., Gruenewald, S. M., Larcos, G. & Jamali, M. Neonatal fungal ventriculitis. *J. Clin. Ultrasound* **34**, 402–406 (2006).](http://paperpile.com/b/W31M7G/YT98)

154.	[de Mol, P., Laureys, W. & Dorchy, H. Neonatal meningitis due to Candida albicans, associated with osteomyelitis; failure to respond to miconazole. *J. Infect.* **5**, 195–197 (1982).](http://paperpile.com/b/W31M7G/4aLu)

155.	[Lin, D. J., Sacks, A., Shen, J. & Lee, T. C. Neurocandidiasis: a case report and consideration of the causes of restricted diffusion. *J. Radiol. Case Rep.* **7**, 1–5 (2013).](http://paperpile.com/b/W31M7G/HRQI)

156.	Iwashita, Hiroshi, *et al.* Occurrence of Candida-specific oligoclonal IgG antibodies in CSF with Candida meningoencephalitis. **579-581**(1978).

157.	[Maschke, M. *et al.* Opportunistic CNS infection after bone marrow transplantation. *Bone Marrow Transplant.* **23**, 1167–1176 (1999).](http://paperpile.com/b/W31M7G/UiDp)

158.	[Jeantin, L. *et al.* Pearls & oy-sters: Spinal cord candidiasis linked to CARD9 deficiency masquerading as a longitudinally extensive transverse myelitis. *Neurology* **99**, 475–479 (2022).](http://paperpile.com/b/W31M7G/H6o2)

159.	[Kuzdan, C. *et al.* Persistent candida albicans infection of A cerebrospinal fluid shunt infection unresponsive to amphotericin B treatment because of increased minimum inhibitory concentration. *Çocuk Enfeksiyon Derg./J. Pediatr. Infect.* **9**, 181–184 (2015).](http://paperpile.com/b/W31M7G/s8sO)

160.	[Puertas Sanjuan, A., Parramón-Teixidó, C. J., Hernandez-Perez, S., Frick, M. A. & Cabañas Poy, M. J. Persistent dyselectrolytemia in a neonate induced by liposomal amphotericin B. A case report. *Front. Pediatr.* **10**, 1099305 (2022).](http://paperpile.com/b/W31M7G/uvva)

161.	[Lin, J.-J., Wu, C.-T., Hsia, S.-H., Wang, H.-S. & Lin, K.-L. Pneumocephalus: a rare presentation of Candida sphenoid sinusitis. *Pediatr. Neurol.* **40**, 398–400 (2009).](http://paperpile.com/b/W31M7G/OZT6)

162.	[Miranda, M. A., Sousa, S. C. & Montes, V. L. Post-COVID-19 neurocandidiasis. *Neurol. Sci.* **42**, 4419–4420 (2021).](http://paperpile.com/b/W31M7G/1zD0)

163.	[Mason, T. B., 2nd *et al.* Postinflammatory hydrocephalus and intracranial mass lesion from Candida in an immunocompetent child. *J. Child Neurol.* **11**, 336–341 (1996).](http://paperpile.com/b/W31M7G/SFfL)

164.	[Leggiadro, R. J. & Collins, T. Postneurosurgical Candida lusitaniae meningitis. *Pediatr. Infect. Dis. J.* **7**, 368–369 (1988).](http://paperpile.com/b/W31M7G/i35o)

165.	[Pahud, B. A., Greenhow, T. L., Piecuch, B. & Weintrub, P. S. Preterm neonates with candidal brain microabscesses: a case series. *J. Perinatol.* **29**, 323–326 (2009).](http://paperpile.com/b/W31M7G/LjIe)

166.	[Huttova, M. *et al.* Prospective study of nosocomial fungal meningitis in children--report of 10 cases. *Scand. J. Infect. Dis.* **30**, 485–487 (1998).](http://paperpile.com/b/W31M7G/Ej63)

167.	[DeJonge, M. H. Radiological case of the month. Candida meningitis in a low-birth-weight infant. *Arch. Pediatr. Adolesc. Med.* **151**, 845–846 (1997).](http://paperpile.com/b/W31M7G/dVg1)

168.	[McAbee, G. *et al.* Rapid and fatal neurologic deterioration due to central nervous system Candida infection in an HIV-1-infected child. *J. Child Neurol.* **10**, 405–407 (1995).](http://paperpile.com/b/W31M7G/cRy0)

169.	[Guo, Y., Wang, P., Lu, L., Xia, S. & Shen, W. Rare appearance of Candida tropicalis infection of the brain: Multiple micro-abscesses combined with diffuse hemorrhages. *Radiol. Infect. Dis.* **1**, 33–36 (2014).](http://paperpile.com/b/W31M7G/zxDI)

170.	[Bozynski, M. E., Naglie, R. A. & Russell, E. J. Real-time ultrasonographic surveillance in the detection of CNS involvement in systemic Candida infection. *Pediatr. Radiol.* **16**, 235–237 (1986).](http://paperpile.com/b/W31M7G/Yto7)

171.	[Dawson, N. L., Robles, H. A. & Alvarez, S. Recurrent Candida tropicalis meningitis. *Clin. Neurol. Neurosurg.* **107**, 243–245 (2005).](http://paperpile.com/b/W31M7G/o6Ly)

172.	[Turhan, V., Acar, A., Çoban, M., Öncül, O. & Çavuşlu, Ş. Recurrent gastrointestinal bleeding, intraabdominal abscesses and synchronous candidemia and meningitis due to leflunomide therapy: Case report. *Turk. Klin. J. Med. Sci.* **30**, 2027–2030 (2010).](http://paperpile.com/b/W31M7G/0kIU)

173.	[Liu, K.-H. *et al.* Refractory candidal meningitis in an immunocompromised patient cured by caspofungin. *J. Clin. Microbiol.* **42**, 5950–5953 (2004).](http://paperpile.com/b/W31M7G/5MI3)

174.	[Yang, S.-H., Iao, K., Huang, A. H. & Wang, K. Ruptured infective intracranial aneurysm with intracerebral hemorrhage in an infant. *J. Med. Sci.* **35**, 47 (2015).](http://paperpile.com/b/W31M7G/O7ta)

175.	[Queiroz, L. de S., Nucci, A. & Faria, J. L. de. Candidíase sistêmica com localização encefálica: estudo anátomo-clínico de cinco casos. *Arq. Neuropsiquiatr.* **34**, 18–31 (1976).](http://paperpile.com/b/W31M7G/Y7en)

176.	[Auriti, C. *et al.* Shunt lock therapy with micafungin to treat shunt-associated Candida albicans meningitis in an infant. *J. Antimicrob. Chemother.* **71**, 2060–2061 (2016).](http://paperpile.com/b/W31M7G/VQGg)

177.	[Xu, L., Zhao, H., Zhou, M., Lang, G. & Lou, H. Single and repeated episodes of Candida species isolated from cerebrospinal fluid for diagnosing probable Candida meningitis. *Front. Microbiol.* **12**, 742931 (2021).](http://paperpile.com/b/W31M7G/YZXY)

178.	[Mirhendi, H. *et al.* South Asian (Clade I) Candida auris meningitis in a paediatric patient in Iran with a review of the literature. *Mycoses* **65**, 134–139 (2022).](http://paperpile.com/b/W31M7G/ghkM)

179.	[Ho, K., Williams, A., Gronseth, G. & Aldrich, M. Spinal cord swelling and candidiasis. A case report. *Neuroradiology* **24**, 117–118 (1982).](http://paperpile.com/b/W31M7G/1JEv)

180.	[Raffa, P. E. A. Z. *et al.* Spinal intramedullary abscess due to Candida albicans in an immunocompetent patient: A rare case report. *Surg. Neurol. Int.* **12**, 275 (2021).](http://paperpile.com/b/W31M7G/sUh5)

181.	[Sakayama, K. *et al.* Subdural spinal granuloma resulting from Candida albicans without immunosufficiency: case report. *Spine (Phila. Pa. 1976)* **27**, E356–60 (2002).](http://paperpile.com/b/W31M7G/rKPP)

182.	[Celmeli, F. *et al.* Successful granulocyte colony-stimulating factor treatment of relapsing Candida albicans meningoencephalitis caused by CARD9 deficiency. *Pediatr. Infect. Dis. J.* **35**, 428–431 (2016).](http://paperpile.com/b/W31M7G/qijZ)

183.	[Chesney, P. J., Teets, K. C., Mulvihill, J. J., Salit, I. E. & Marks, M. I. Successful treatment of Candida meningitis with amphotericin B and 5-fluorocytosine in combination. *J. Pediatr.* **89**, 1017–1019 (1976).](http://paperpile.com/b/W31M7G/hT11)

184.	[Behre, G., Christopeit, M. & Weber, T. Successful treatment of Candida spp. encephalitis occurring under Caspofungin treatment after allogeneic stem cell transplantation. *Ann. Hematol.* **88**, 93–94 (2009).](http://paperpile.com/b/W31M7G/ZcWj)

185.	[Singhal, T., Kumar, A., Borade, P., Shah, S. & Soman, R. Successful treatment of C. auris shunt infection with intraventricular caspofungin. *Med. Mycol. Case Rep.* **22**, 35–37 (2018).](http://paperpile.com/b/W31M7G/exCM)

186.	[Phanthumchinda, K. & Kaoropthum, S. Syringomyelia associated with post-meningitic spinal arachnoiditis due to Candida tropicalis. *Postgrad. Med. J.* **67**, 767–769 (1991).](http://paperpile.com/b/W31M7G/c6xF)

187.	[Esposito, P., Zerr, N., Kehrli, P., Pain, L. & Maitrot, D. Complication neuroméningée d’une infection systémique à Candida albicans chez un prématuré. *Rev. Neurol. (Paris)* **161**, 832–835 (2005).](http://paperpile.com/b/W31M7G/JyYP)

188.	[Faix, R. G. Systemic Candida infections in infants in intensive care nurseries: high incidence of central nervous system involvement. *J. Pediatr.* **105**, 616–622 (1984).](http://paperpile.com/b/W31M7G/s2Sr)

189.	[Tonami, N. *et al.* Thallium-201 accumulation in cerebral candidiasis: unexpected finding on SPECT. *Clin. Nucl. Med.* **15**, 397–400 (1990).](http://paperpile.com/b/W31M7G/S3G6)

190.	[Parker, J. C., Jr, McCloskey, J. J. & Lee, R. S. The emergence of candidosis. The dominant postmortem cerebral mycosis. *Am. J. Clin. Pathol.* **70**, 31–36 (1978).](http://paperpile.com/b/W31M7G/MItC)

191.	[Andrew, N. H., Ruberu, R. P. & Gabb, G. The first documented case of Candida dubliniensis leptomeningeal disease in an immunocompetent host. *BMJ Case Rep.* **2011**, bcr0620114384 (2011).](http://paperpile.com/b/W31M7G/RD9N)

192.	[Murphy, K., Bradley, J. & James, H. E. The treatment of Candida albicans shunt infections. *Childs. Nerv. Syst.* **16**, 4–7 (2000).](http://paperpile.com/b/W31M7G/7IXf)

193.	[Morison, A., Erasmus, D. S. & Bowie, M. D. Treatment of Candida albicans meningitis with intravenous and intrathecal miconazole. A case report. *S. Afr. Med. J.* **74**, 235–236 (1988).](http://paperpile.com/b/W31M7G/shSO)

194.	[Mendes, V., Castro, S., Linhares, P. & Ribeiro-Silva, M. L. Tumoriform presentation of cerebral candidiasis in an HIV-infected patient. *J. Clin. Neurosci.* **16**, 587–588 (2009).](http://paperpile.com/b/W31M7G/a0js)

195.	[Mahboubi, S., Kaufmann, H. J. & Schut, L. Two instances of inflammatory aqueductal occlusion in prematures after neonatal candidal septicemia. *Clin. Pediatr. (Phila.)* **15**, 651–653 (1976).](http://paperpile.com/b/W31M7G/TETT)

196.	[Kirpekar, M., Abiri, M. M., Hilfer, C. & Enerson, R. Ultrasound in the diagnosis of systemic candidiasis (renal and cranial) in very low birth weight premature infants. *Pediatr. Radiol.* **16**, 17–20 (1986).](http://paperpile.com/b/W31M7G/vuUX)

197.	[Hurlbert, R. J., Bilbao, J. M. & Tucker, W. S. ‘Unexplained’ delayed death from fungal meningitis after meningioma resection. *Can. J. Neurol. Sci.* **22**, 239–243 (1995).](http://paperpile.com/b/W31M7G/HiOY)

198.	[Cao, R.-L. Unexplained seizures after intestinal surgery. *Pediatr. Neurol.* **51**, 587–588 (2014).](http://paperpile.com/b/W31M7G/JCGX)

199.	[Lisch, S. & Steudel, W. I. Ungewöhnlicher Verlauf einer Candidiasis des Zentralnervensystems. *Dtsch. Med. Wochenschr.* **119**, 13–18 (1994).](http://paperpile.com/b/W31M7G/06Zr)

200.	[Cruciani, M. *et al.* Use of fluconazole in the treatment of Candida albicans hydrocephalus shunt infection. *Eur. J. Clin. Microbiol. Infect. Dis.* **11**, 957 (1992).](http://paperpile.com/b/W31M7G/1mAF)

201.	[Grimes, D. A., Lach, B. & Bourque, P. R. Vasculitic basilar artery thrombosis in chronic Candida albicans meningitis. *Can. J. Neurol. Sci.* **25**, 76–78 (1998).](http://paperpile.com/b/W31M7G/7N6h)

202.	[Jamjoom, A. *et al.* Ventriculitis and hydrocephalus caused by Candida albicans successfully treated by antimycotic therapy and cerebrospinal fluid shunting. *Br. J. Neurosurg.* **6**, 501–504 (1992).](http://paperpile.com/b/W31M7G/yJ3J)

203.	[Tsakiri, S. *et al.* Voriconazole treatment for an infant with intractable Candida glabrata meningitis. *Pediatr. Infect. Dis. J.* **37**, 999–1001 (2018).](http://paperpile.com/b/W31M7G/kbKf)

204.	[Ceccarelli, G. *et al.* Voriconazole treatment of Candida tropicalis meningitis: persistence of (1,3)-β-D-glucan in the cerebrospinal fluid is a marker of clinical and microbiological failure: A case report. *Medicine (Baltimore)* **95**, e4474 (2016).](http://paperpile.com/b/W31M7G/Z5DD)

205.	[Gülez, N. & Genel, F. Candida albicans meningitis associated with intracranial hemorrhage. *Marmara Medical Journal* **20**, 37–42 (2007).](http://paperpile.com/b/W31M7G/yrxU)

206.	[Nordström, L., Oistämö, S. & Olmebring, F. Candida meningoencephalitis treated with 5-fluorocytosine. *Scand. J. Infect. Dis.* **9**, 63–64 (1977).](http://paperpile.com/b/W31M7G/GG0c)

207.	[Gorell, J. M., Palutke, W. A. & Chason, J. L. Candida pachymeningitis with multiple cranial nerve pareses. *Arch. Neurol.* **36**, 719–720 (1979).](http://paperpile.com/b/W31M7G/Yc9y)

208.	[Chattopadhyay, B. Candida tropicalis meningitis: a case report. *J. Laryngol. Otol.* **95**, 1149–1151 (1981).](http://paperpile.com/b/W31M7G/xDBq)

209.	[Henig, E., Djaldetti, M., Pinkhas, J. & De Vries, A. Candida tropicalis meningitis in Hodgkin’s disease. *JAMA* **199**, 214–215 (1967).](http://paperpile.com/b/W31M7G/MgeX)

210.	[Sarmiento, R., Holubka, J. & Khatib, R. Case report: Candida meningitis with an intradural filling defect 1 year after candidemia. *Am. J. Med. Sci.* **307**, 115–118 (1994).](http://paperpile.com/b/W31M7G/5CqT)

211.	[de Medeiros, B. C. *et al.* Central nervous system infections following bone marrow transplantation: an autopsy report of 27 cases. *J. Hematother. Stem Cell Res.* **9**, 535–540 (2000).](http://paperpile.com/b/W31M7G/qNGT)

212.	[Benjamin, L. A. *et al.* Diagnostic accuracy of the Recognition of Stroke in the Emergency Room (ROSIER) score and CT brain in an HIV population. *J. Infect.* **67**, 619–622 (2013).](http://paperpile.com/b/W31M7G/G9z1)

213.	[Moran, C. A., Terry, C., Nguyen, M. L. T. & Sheth, A. N. Use of cerebrospinal fluid (1,3)-β-d-glucan to monitor treatment response in Candida albicans meningitis in an HIV-infected patient. *Infect. Dis. Clin. Pract. (Baltim. Md.)* **26**, e70–e72 (2018).](http://paperpile.com/b/W31M7G/EY46)

214.	[Askari, A. *et al.* Candida dublinensis meningitis in an immunocompetent host: A case report and review of the literature. *Neurol. Clin. Pract.* **14**, e200279 (2024).](http://paperpile.com/b/W31M7G/e1Io)

215.	[Huang, C. C. *et al.* Central nervous system candidiasis in very low-birth-weight premature neonates and infants: US characteristics and histopathologic and MR imaging correlates in five patients. *Radiology* **209**, 49–56 (1998).](http://paperpile.com/b/W31M7G/EJt3)

216.	[Kantor, H. L., Emsellem, H. A., Hogg, J. E. & Simon, G. L. Candida albicans meningitis in a parenteral drug abuser. *South. Med. J.* **77**, 404–405 (1984).](http://paperpile.com/b/W31M7G/riJq)

217.	[Anhalt, E., Alvarez, J. & Berg, R. Candida glabrata meningitis. *South. Med. J.* **79**, 916 (1986).](http://paperpile.com/b/W31M7G/VT05)

218.	[Rao, H. K. & Myers, G. J. Candida meningitis in the newborn. *South. Med. J.* **72**, 1468–1471 (1979).](http://paperpile.com/b/W31M7G/8vXB)

219.	[White, B. E. Cerebral candidiasis. *N. Engl. J. Med.* **286**, 321 (1972).](http://paperpile.com/b/W31M7G/1Kr0)

220.	[Binning, M. J., Lee, J., Thorell, E. A. & Riva-Cambrin, J. K. Intraventricular fungus ball: a unique manifestation of refractory intracranial candidiasis in an immunocompetent neonate. *J. Neurosurg. Pediatr.* **4**, 584–587 (2009).](http://paperpile.com/b/W31M7G/KrpN)

221.	[Holyst, J., Majewski, A. & Tyszkiewicz, S. Massive cerebellar abscess due to candida albicans. *Neurochirurgia (Stuttg.)* **19**, 126–129 (1976).](http://paperpile.com/b/W31M7G/j3bO)

222.	[Zimmerman, S. L., Frutchey, L. & Gibbes, J. H. Meningitis due to Candida (Monilia) albicans with recovery. *J. Am. Med. Assoc.* **135**, 145–147 (1947).](http://paperpile.com/b/W31M7G/igTb)

223.	[Guerreiro, R., Rodrigues, M., Fontoura, P. & Pinto-Marques, J. Multiple brain abscesses of a mycotic origin in an immunocompetent individual. *Rev. Neurol.* **43**, 567–568 (2006).](http://paperpile.com/b/W31M7G/IlOj)

224.	[Halpert, B. & Wilkins, H. Mycotic meningitis due to Candida. *J. Am. Med. Assoc.* **130**, 932–934 (1946).](http://paperpile.com/b/W31M7G/WrlP)

225.	[Xu, W., Liu, C. F., Xue, C. L. & Lin, N. Onset of meningoencephalitis: disseminated candidiasis. *Pediatr. Int.* **53**, 1102–1103 (2011).](http://paperpile.com/b/W31M7G/eiCY)

226.	[Maggiore, G., Marseglia, G. & Duse, M. Septicemia and meningitis as a complication of liver biopsy in a girl with chronic mucocutaneous candidiasis. *J. Pediatr. Gastroenterol. Nutr.* **1**, 614 (1982).](http://paperpile.com/b/W31M7G/dcXZ)

227.	[Scott, P. A. & Ohlsson, A. Sequelae associated with cerebral candidiasis in two premature infants. *Acta Paediatr.* **87**, 1090–1092 (1998).](http://paperpile.com/b/W31M7G/JKZe)

228.	[Ehni, W. F. & Ellison, R. T., 3rd. Spontaneous Candida albicans meningitis in a patient with the acquired immune deficiency syndrome. *Am. J. Med.* **83**, 806–807 (1987).](http://paperpile.com/b/W31M7G/IPUN)

229.	[Kumar, S., Kaza, R. C., Tandon, A. & Nayar, M. Subdural spinal fungal granuloma due to Candida tropicalis. Case report. *J. Neurosurg.* **50**, 395–396 (1979).](http://paperpile.com/b/W31M7G/kmg0)

230.	[Lenoir, P., De Meirleir, L., Bougatef, A., Marchand, J. & Sacré, L. Unexpected diagnosis of Candida albicans meningitis in a premature neonate. *Pediatr. Neurol.* **5**, 370–372 (1989).](http://paperpile.com/b/W31M7G/R4ni)

231.	[Ponomarev, V. V. & Rakot, T. A. Clinical case of hypertrophic convexital pachymeningitis. *Russian neurological journal* **28**, 55–60 (2023).](http://paperpile.com/b/W31M7G/v3jq)

232.	[Bashall, N. & Aggarwal, A. 523: Intrathecal caspofungin: A last resort therapy for resistant candida auris ventriculitis. *Crit. Care Med.* **52**, S235–S235 (2024).](http://paperpile.com/b/W31M7G/VyfK)

233.	[Fine, J. M., Franklin, D. A. & Lieberthal, A. S. Mycotic meningitis due to candida albicans; a four year recovery. *Neurology* **5**, 438–443 (1955).](http://paperpile.com/b/W31M7G/XjHM)

234.	[Ferrari, V. & Mazzanti, C. P480 submitral left ventricular aneurysm and stroke in a young Caucasian woman. *Eur. Heart J. Suppl.* **25**, D231–D231 (2023).](http://paperpile.com/b/W31M7G/fx82)

235.	[Granda Lobato, P. *et al.* 4CPS-054 Use of intrathecal liposomal-amphotericin B for Candida meningitis: a case report. in *Section 4: Clinical pharmacy services* (British Medical Journal Publishing Group, 2023). doi:](http://paperpile.com/b/W31M7G/MkX1)[10.1136/ejhpharm-2023-eahp.89](http://dx.doi.org/10.1136/ejhpharm-2023-eahp.89)[.](http://paperpile.com/b/W31M7G/MkX1)

236.	[Toprak, D. *et al.* Recurrent Candida albicans ventriculitis treated with intraventricular liposomal amphotericin B. *Case Rep. Infect. Dis.* 340725 (2015).](http://paperpile.com/b/W31M7G/iNuq)

237.	[Mori, T. & Ebe, T. Analysis of cases of central nervous system fungal infections reported in Japan between January 1979 and June 1989. *Intern. Med.* **31**, 174–179 (1992).](http://paperpile.com/b/W31M7G/w0WH)

238. Crause, C. A case of disseminated Candida dubliniensis in a preterm infant: The importance of early detection and management of invasive fungal infections in neonates. *South African Journal of Child Health*. Jan 1;9(1):27-9 (2015).

239. Letournel, F. *et al.* A fatal Candida albicans meningoencephalomyeloradiculitis in an immunocompetent patient: case report and review of the literature: Po15-2. Brain Pathology 20:82 (2010).

240. Raja, P., and Mohtarrudin, N. *Disseminated Candidiasis with Cerebral Involvement in a Neonate.* *Journal of Pathology.*13th Joint Meeting of the British Division of the International Academy of Pathology and the Pathological Society of Great Britain & Ireland (2021).

241. Edmin, W. and Finlayson, M. H. Moniliasis of the central nervous system in a child with recover*y. South African Medical Journal* 28.41:868-871 (1954).

242. Hoshide, R. R. *et al.* A case of Candida ventriculitis in a patient with an antibiotic-impregnated external ventricular drain. Do antibiotic-impregnated external ventricular drains confer a new selection of micro-organisms? *Neurocrit Care.*19:S1–S33 (2013).

243. Ozsvath, B., and M. de Wit. Candida kefyr caused fungal pneumonia, brain abscesses and multiorgan failure in a patient with acute myeloid leukemia. *Onkologie*. 2011;34(suppl 6):1–305 (2011).

244. Chen, J. *et al.* Caspase recruitment domain-containing protein 9 gene deficiency associated invasive candidiasis: a case report and literature review. *Chinese Journal of Neurology.* Vol. 54, No. 4; 355-36 (2021).

245. Maximova, N. *et al.* Highly resistant Candida cerebral infection. *Mycoses*. Vol. 55. (2012).

246. Adachi, T., Hoshino, H.,. and Arakawa, C.. Rapid progressive intracranial aneurysm resulting from Candida infective endocarditis. *International Journal of Stroke*. Vol. 13. (2018).

247. Vinh, D. C. Recurrent spontaneous central nervous system candidiasis: what to do when the cards are stacked against you?. *Journal of Clinical Immunology.* Vol. 34. No. 3. 233 (2014).

248. Goudjil, S. *et al.* Regression of infectious-suspected brain lesion in a premature infant during treatment of invasive candida infection with echinocandins. *The Journal of Maternal-Fetal & Neonatal Medicine.* 29:sup1, 1-313 (2016).

249. Rocha, R. *et al*. Neurologic complications of fungic endocarditis. *European Journal of Neurology.* Vol. 28. (2021).
